# Cowpea genetic diversity, population structure and genome-wide association studies in Malawi: insights for breeding programs

**DOI:** 10.3389/fpls.2024.1461631

**Published:** 2025-01-20

**Authors:** Michael M. Chipeta, John Kafwambira, Esnart Yohane

**Affiliations:** ^1^ Department of Crop and Soil Sciences, Lilongwe University of Agriculture and Natural Resources, Lilongwe, Malawi; ^2^ Department of Agricultural Research Services, Chitedze Research Station, Lilongwe, Malawi

**Keywords:** clustering, cowpea breeding, DArTag single-nucleotide polymorphism, genetic variability, morphological characterization, principal component analysis, marker trait association

## Abstract

**Introduction:**

This study focuses on cowpea, a vital crop for smallholder farmers in sub-Saharan Africa, particularly in Malawi. The research aimed to understand the genetic diversity and population structure of cowpea and to perform genome-wide association studies (GWAS) to identify marker-trait associations (MTAs) for yield and related traits. These insights are intended to support varietal development and address agricultural challenges in Malawi.

**Methods:**

A total of 306 cowpea genotypes were characterized using single nucleotide polymorphism (SNP) markers and morphological traits. The study assessed the effects of genotype, location, and their interactions on morphological traits. The Fixed and Random Model Circulating Probability Unification (FarmCPU) algorithm was used to identify significant MTAs.

**Results:**

The morphological traits showed significant genotype, location, and interaction effects. Genotypes MWcp24, MWcp47, MWcp2232, and TVu-3524 yielded the highest values. Grain yield was positively correlated with peduncle length, seeds per pod, and pods per plant. Three distinct clusters were identified based on morphological traits. Genetic diversity analysis revealed an average minor allele frequency of 0.31, observed heterozygosity of 0.06, and gene diversity of 0.33. The average inbreeding coefficient was 0.82, indicating a high level of inbreeding. Most of the genetic variation (73.1%) was found among genotypes within populations. Nine groups and ancestral populations were identified, which did not entirely overlap with geographic origins. Sixteen significant MTAs were linked to six morphological traits.

**Discussion:**

The validation of these identified MTAs, along with the observed genetic diversity, offers valuable opportunities for cowpea improvement through marker-assisted selection, to addresses the challenges faced by Malawian farmers. The identification of thirty cowpea lines as key founder lines for breeding programs in Malawi, Mozambique, and Tanzania is a significant outcome. These efforts aim to develop more productive cowpea lines for the region, enhancing food security and agricultural sustainability.

## 1 Introduction

Cowpea (*Vigna unguiculata*), a versatile and resilient annual grain legume, is grown across the globe but it holds particular significance in sub-Saharan Africa, where it plays a critical role in food security and rural livelihoods (Boukar et al., 2019). Cowpea is not only a vital income source but also a critical component of both human and animal nutrition for millions of smallholder farmers and consumers in the region (Omomowo and Babalola, 2021). The nutrient-packed grains are rich in protein (25%) and contain essential minerals such as zinc (38.1 mg/kg), iron (53.2 mg/kg), magnesium (1915 mg/kg), calcium (826 mg/kg), potassium (14,890 mg/kg), and phosphorus (5055 mg/kg) (Boukar et al., 2011; International Institute of Tropical Agriculture (IITA), 2016). Beyond its nutritional value, cowpea enhances soil fertility through biological nitrogen fixation and serves as an effective cover crop, contributing to sustainable farming practices (Alemu et al., 2016; International Institute of Tropical Agriculture (IITA), 2016).

As of 2022, global cowpea production was estimated at 9.8 million metric tons (MT) from 15.2 million hectares with an average yield of 0.64 tons per hectare (t/ha). Africa accounted for most of this production, contributing approximately 9.5 million MT from 14.9 million hectares. West Africa leads the continent in cowpea production, with Nigeria and Niger as the top producers, while other countries like Burkina Faso, Cameroon, Ghana, Kenya, Uganda, and Tanzania also produce significant quantities of cowpea. In contrast, Malawi’s cowpea production in 2022 was limited to just 0.05 million MT from 1.02 million hectares (FAOSTAT, 2024).

Despite the significant area cultivated, Malawi’s cowpea productivity lags behind the global average, at 350 kg/ha. The lower yield is primarily due to factors such as abiotic stresses (such as drought, heat, and low soil fertility), biotic stresses (such as insect pests, diseases, and parasitic weeds), and poor crop management practices. Moreover, the continued reliance on landraces, coupled with the lack of improved varieties that meet farmer preferences, exacerbates the situation (Coulibaly et al., 2010; Gomes et al., 2019; Hella et al., 2013; Nkhoma et al., 2020; Nkongolo et al., 2009). Therefore, addressing these challenges by developing improved, high-yielding varieties is essential for enhancing productivity, meeting market demand, and unlocking the full potential of cowpea in Malawi.

Cowpea is a diploid species (2n = 22) with a genome size of approximately 620 Mbp (Boukar et al., 2018; Lonardi et al., 2019; Timko et al., 2008). Although the crop is primarily self-pollinating, it exhibits an outcrossing rate of up to 5% (Badiane et al., 2014). Archaeological evidence from Ghana suggests cowpea was domesticated around 1500 BC (D’Andrea et al., 2007) although the primary center of domestication remains debated. Many taxonomists suggest West Africa as the primary center of domestication (Kouam et al., 2012), while others propose India and Southeast Asia following its introduction from Africa.

The cultivated cowpea belongs to the subspecies *unguiculata*, with its center of diversity in West and Central Africa (Padulosi and Ng, 1997). This subspecies has five cultivar groups: Unguiculata, Sesquipedalis, Textilis, Biflora, and Melanophthalmus (Pasquet, 1998; 2000). In contrast, the center of diversity for wild cowpea relatives is found in southern Africa, stretching across Namibia, Botswana, Zambia, Zimbabwe, Mozambique, South Africa, and Swaziland.

Genetic diversity assessment is a critical component of breeding programs aimed at developing improved varieties. It broadens the genetic base for breeding and facilitates the identification and selection of desirable parental lines (Prasanthi et al., 2012). Traditional methods of assessing genetic diversity in cowpea based on morphological markers (phenotypic traits), have been widely documented (Boukar et al., 2011; Gerrano et al., 2015; Govindaraj et al., 2015; Mafakheri et al., 2017; Muranaka et al., 2015; Nkhoma et al., 2020; Stoilova and Pereira, 2013). While these markers offer insights into morphological and physiological variation, they are greatly influenced by environmental factors, vary across developmental stages, and are limited in number, which can restrict their ability to fully capture genetic diversity (De Vicente and Fulton, 2003).

Advances in genomics have significantly improved the study of genetic diversity, particularly through the use of molecular markers (Boukar et al., 2019). Tools such as random amplified polymorphic DNA (RAPD), restriction fragment length polymorphism (RFLP), amplified fragment length polymorphism (AFLP), simple sequence repeat (SSR) and Single Nucleotide Polymorphisms (SNP) have proven invaluable for dissecting genetic diversity and population structure in cowpea (Boukar et al., 2016; Guimarães et al., 2023; Gumede et al., 2022; Nkhoma et al., 2020; Prasanthi et al., 2012). Despite their precision, molecular markers may not directly correlate with agronomic traits, hence the need to combine them with morphological markers to draw meaningful conclusions (Gumede et al., 2022).

Genome-Wide Association Studies (GWAS) provide a powerful genomic tool that integrates phenotypic and genomic data to identify candidate loci associated with specific traits, which can then be validated in follow-up studies (Uffelmann et al., 2021). GWAS which is based on linkage disequilibrium (LD) formed in populations over several generations, utilizes germplasm from diverse backgrounds shaped by several historical and ancestral recombination events (Mackay and Powell, 2007). The approach helps in understanding genetic basis for traits like grain yield and its components, enabling the capture of superior alleles that have been missed by breeding practices. Grain yield is a complex quantitative trait, governed by numerous genes with small individual effects and is further complicated by significant genotype × environment interactions (Santos et al., 2024). While molecular breeding holds promise for improving such a complex trait, a comprehensive understanding of its genetic architecture is crucial for the effective application of molecular tools.

Recently, the Diversity Arrays Technology (DArT) platform has become a more accessible and cost-effective tool for genetic analysis. DArT has been successfully applied to study genetic diversity, population structure, and GWAS in cowpea, using DArTseq SNP markers (Abberton et al., 2024; Koura et al., 2024; Edema et al., 2023; Ketema et al., 2020; Nkhoma et al., 2020). While many studies have investigated genetic diversity and marker-trait associations (MTAs) using these and other markers, further research is needed using different genetic materials and cost-effective markers, as we have not yet reached a point of saturation.

DArT introduced targeted genotyping (DArTag-a medium-density marker panel) that can target any SNP (or a small indel), provided there is some genomic sequence around the variant base/indel. The platform is accredited for being a cost-efficient approach that reduces bioinformatics load and is well-suited for high-throughput scenarios (Ongom et al., 2024). The Cowpea mid-density genotyping panel includes 2,602 SNP markers, averaging approximately 3 SNPs per centimorgan (cM) across the 11 cowpea chromosomes. Ongom et al. (2024) confirmed its accuracy in assessing genetic relatedness, diversity, and population structure.

Given the challenges faced by farmers in Malawi, the development of improved cowpea varieties with enhanced traits, particularly those that address the specific challenges requires continued innovation in genomics and breeding methodologies. Hence, this study was designed to (i) assess the genetic diversity and population structure of cowpea germplasm in Malawi, using both morphological traits and medium-density DArTag marker panel and (ii) conduct GWAS analysis for yield and related traits to identify the MTAs to deploy in marker assisted selection (MAS). The goal is to guide the development of improved cowpea varieties with demand-driven traits in Malawi.

## 2 Materials and methods

### 2.1 Plant materials

Study materials comprised 306 diverse cowpea genotypes (**Supplementary Table S1**) sourced from cowpea farmers in Malawi, Mozambique, and Tanzania, and from the International Institute for Tropical Agriculture (IITA) gene bank in Ibadan, Nigeria. In terms of their origin, the genotypes were categorized as from Nigeria genebank (45), Mozambique (23), Malawi (78), Ghana (2), Nigeria (13), Philippines (1), South Africa (9), USA (21), Hungary (2), Uganda (4), Argentina (1), India (61), USSR (2), Zambia (4), Senegal (2), Cameroon (2), Botswana (2), Zimbabwe (1), Niger (1), Benin (1), Italy (1) and Tanzania (16). Additionally, 14 genotypes were of unknown origin. The distribution of genotypes across regions was as follows: Southern Africa (38.2%), West Africa (20.9%), Asia (20.3%), America (7.2%), East Africa (65.5%), unknown origin (4.6%), Europe (1.6%) and Central Africa (0.7%).

### 2.2 Description of experimental sites, experimental protocol, morphological data collection and data analysis

Field trials were established at Bunda College of Agriculture of Lilongwe University of Agriculture and Natural Resources and Chitedze Research Station in Malawi in the 2021/2022 growing season. Bunda College of Agriculture is located between latitude 14˚11’S and longitude 33˚46’E at an altitude of 1100 meters above sea level while Chitedze Research Station is located between latitude 13° 59’ S and longitude 33° 38’ E at an altitude of 1146 meters above sea level. Field trials were conducted using an incomplete block design using 34 blocks of size 9 (9 × 34) with 3 replications at each site. Plot sizes were of 2 m long ridges spaced at 75 cm. Two seeds were planted at each planting station spaced 20 cm. Field management followed the recommended cowpea agricultural practices. Data was collected for nine morphological traits: peduncle length, days to 50% flowering, days to 90% maturity, pods per plant, seeds per pod, seed length, seed width, 100-seed weight, and grain yield (**Supplementary Table S2**), as specified by the International Board for Plant Genetic Resources (International Board for Plant Genetic Resources, 1983).

The phenotypic data for the morphological traits were initially analyzed separately for each location by fitting a linear mixed model for each location to obtain location-specific best linear unbiased estimates (BLUEs) and variance components. The model was specified as follows:


$$Y_{ijk}=µ+Rep_{i}+Block_{j}\left(Rep_{i}\right)+Gen_{k}+e_{ij}$$

where *Y_ijk_* represents the trait of interest, *µ* is the overall mean effect, *Rep_i_* is the effect of the i^th^ replicate, *Block_j_* is the effect of the j^th^ incomplete block within the i^th^ replicate, Gen_k_ is the effect of the k^th^ genotype and *e_ijk_* is the error term. Following this, a combined analysis across the two locations was conducted to estimate both BLUEs and best linear unbiased predictions (BLUPS) by adjusting the statistical model as follows:


$$Y_{ijkl}=µ+Loc_{i}+Rep_{j}\left(Loc_{i}\right)+Block_{k}\left(Loc_{i}Rep_{j}\right)+Gen_{l}+Loc_{1}\times Gen_{l}+e_{ij}$$

Here, the new terms *Loc_i_* and *Loc_i_ × Gen_l_* represent the effects of the i^th^ environment and the environment × genotype *(G × E)* interaction, respectively. In the model, all effects, except the overall mean were treated as random. The analyses were performed using *META-R* (Alvarado et al., 2020) and the *LME4 R* package (Bates et al., 2015).

Using the BLUPs, Pearson’s correlation analysis was conducted to explore associations among the traits, employing the *corr_coef* function from the *metan* package in R (Olivoto and Lúcio, 2020). To further investigate variations among genotypes, principal component analysis (PCA) was performed using the *FactoMineR* (Lê et al., 2008) and *factoextra* (Kassambara and Mundt, 2017) packages. Additionally, hierarchical clustering on the principal components (HCPC) was carried out to identify genotype groupings based on shared attributes.

### 2.3 SNP genotyping and quality control

Samples for genotyping were obtained from seedlings of the cowpea genotypes that were raised in pots in a screen house at Bunda College of Agriculture. A leaf from each labeled plant was collected for DNA analysis three weeks post-germination. This collection process adhered to the protocol established by the Intertek-Agritech laboratory (Intertek-Agritech, 2016). The genomic DNA was then extracted at the Intertek laboratory in Sweden. Following this, the samples were dispatched to the Diversity Arrays Technology (DArT) facility in Canberra, Australia, where they underwent genotyping using the DArTag technology.

The DArTag dataset initially contained 2,753 SNPs. After quality control and filtering using PLINK 1.9 (Chang et al., 2015), 2428 SNP high-quality markers and 305 genotypes remained for downstream analysis. The filtering process removed markers with 15% missing data, minor allele frequencies (MAF) less than 0.05, and one genotype (TVu-1178) with a missing data rate exceeding 10%. Linkage disequilibrium (LD) estimates between marker pairs were obtained using TASSEL version 5.0 (Bradbury et al., 2007). The pairwise LD values (r^2^) were plotted against genetic distance and fitted with a LOESS regression curve using R statistical package (R Core Team, 2021). The pattern of LD decay was determined where the LOESS regression of mean r^2^ between pairs of SNPs intercepted the threshold of 0.2

### 2.4 Genetic diversity

Using the filtered SNP dataset, a genlight object was created in R version 4.1.2 (R Core Team, 2021) with the dartR package (Gruber et al., 2018) and its dependency, adegenet (Jombart, 2008). The object combined marker data, SNP metrics, and population metadata. The quality of the markers was further assessed based on minor allele frequency, call rate and polymorphic information content (PIC) using the dartR package. The package was also used to estimate genetic diversity parameters; observed heterozygosity (Ho), gene diversity (He), inbreeding coefficients (Fis), and minor allele frequency (MAF) for predefined subpopulations based on geographic origins. Additionally, pairwise Nei’s D genetic distances were computed. Hierarchical clustering was performed using Nei’s distance metrics from the NAM package (Xavier et al., 2015), and group associations were visualized using the ward.D method and hclust function. Finally, a circular dendrogram was constructed using the circlize package (Gu et al., 2024).

### 2.5 Population structure and kinship

Genetic structure among cowpea genotypes was assessed using several methods. First, an analysis of molecular variance (AMOVA) was performed with the poppr package (Kamvar et al., 2014) to quantify genetic variation at three hierarchical levels: between populations, between genotypes within populations, and within genotypes. Next, admixture analysis was conducted using the LEA package (Frichot and François, 2015), which estimates ancestry coefficients from genotypic matrices and identifies ancestral populations (using the snmf function). The optimal number of clusters (K) was determined based on minimum cross-entropy values, and snmf runs were performed for K=2-10. An ancestry matrix was then plotted based on these results. Subsequently, random and fixed effects were estimated to minimize the rate of false positives in GWAS models. The random effects accounted for kinship relationships, while the fixed effects addressed population structure. The kinship matrix was generated using the VanRaden algorithm from the memory-efficient, visualization-enhanced, and parallel-accelerated (rMVP) package (Yin et al., 2021) in R version 4.3.2 (R Core Team, 2023). Population structure further assessed through unsupervised clustering via principal component analysis (PCA), using the same rMVP package version 1.1.1.

### 2.6 Genome-wide association mapping

The GWAS was conducted for all nine traits in the study using the 305 genotypes retained after filtering, with the rMVP package (Yin et al., 2021) in R. The analysis utilized BLUP values for all traits, except for seed width, which used normalized values obtained through log transformation. Trait-SNP associations were identified using the fixed and random model circulating probability unification (FarmCPU) algorithm. FarmCPU is a multilocus model that effectively controls false positives without significantly impacting false negatives (Liu et al., 2016). This algorithm employs a multiple loci linear mixed model to simultaneously include multiple markers as covariates in a step-wise mixed linear model (MLM), addressing confounding issues between covariates and kinship. Any remaining confounding is accounted for by both fixed effect models (FEM) and random effect models (Liu et al., 2016). The first five principal components, calculated by rMVP, were used as covariates to account for population structure. rMVP built-in functions were used to generate Manhattan and QQ plots. The significance threshold for SNP-trait associations was determined using a Bonferroni correction, with a significance level of *P* ≤ 0.05/*n*, where *n* is the total number of SNP markers in the genome.

## 3 Results

### 3.1 Phenotypic variation in cowpea morphological traits

Phenotypic variation based on morphological traits was assessed at two locations (Bunda and Chitedze). The variance components and other statistics for all traits scored at the two locations and across both locations are shown in **Table 1**. Highly significant differences (p < 0.001) were observed for all traits at Bunda location (peduncle length, days to 50% flowering, days to 90% maturity, pods per plant, seeds per pod, seed length, seed width, 100-seed weight, and grain yield). At the Chitedze location, highly significant differences (p < 0.001) were observed for days to 50% flowering, days to 90% maturity, seed length, seed width, and 100-seed weight. Significant differences (p < 0.01) were also found for pods per plant and grain yield while differences for peduncle length and seeds per pod were not significant. Genotype effects (except for seeds per pod), location effects (except for seeds per pod, seed length, seed width, and pods per plant), and the interaction of genotype and location effects (except for seed width and 100-seed weight) showed significant differences (p < 0.05).

**Table 1:** Variance components and statistics for cowpea morphological traits at Bunda College, Chitedze Research Station and across locations.

Statistic	PEDLNG	DF50	MAT90	SDPPOD	SDLEN	SDWDT	PODSPP	HSDWT	GRNYLD
Bunda College of Agriculture									
Genotype Variance	12.03***	44.38***	52.97***	1.97***	1.01***	0.34***	20.89***	10.32***	314063.43***
Residual Variance	18.75	10.04	19.26	2.30	0.59	0.22	30.52	6.89	285861.46
Grand Mean	15.37	61.59	105.25	13.13	7.18	4.94	24.73	12.88	1485.11
Min	8.34	50.06	81.88	9.71	5.44	3.97	12.14	7.31	370.08
Max	22.98	87.53	127.34	16.35	9.84	6.71	37.97	23.66	2813.58
LSD	5.81	5.19	6.79	2.09	1.15	0.70	7.56	3.92	789.04
CV	28.17	5.14	4.17	11.54	10.71	9.57	22.34	20.37	36.00
Chitedze Research Station									
Genotype Variance	0.20ns	7.67***	7.70***	0.08ns	0.32***	0.14***	6.35**	4.93***	126676.41**
Residual Variance	25.27	34.40	42.78	2.07	1.25	0.66	58.49	17.42	995746.37
Grand Mean	27.27	63.19	82.07	14.33	7.21	5.14	26.84	15.88	2674.94
Min	27.05	58.16	78.23	14.06	6.01	4.30	23.20	12.33	2137.24
Max	27.49	69.04	87.77	14.59	8.48	8.48	30.58	21.99	3201.82
LSD	1.23	6.00	6.27	0.76	1.19	0.81	6.15	4.57	848.08
CV	18.43	9.28	7.97	10.04	15.53	15.79	28.49	26.29	37.30
Pooled									
Genotype Variance	3.00***	19.13***	19.22***	0.11ns	0.56***	0.22***	3.47*	7.34***	159744.69***
Gen x Loc Variance	3.15***	7.27***	11.18***	0.89***	0.10**	0.01	10.03***	0.36	59973.68**
Environment Variance	70.14***	1.18*	267.69***	0.65	0.00	0.02	0.00	4.25*	701127.54***
Residual Variance	21.97	21.93	30.89	2.19	0.93	0.45	44.58	11.99	641367.56
Grand Mean	21.32	62.39	93.66	13.73	7.20	5.04	25.79	14.38	2080.09
Min	18.36	54.06	82.39	13.45	5.83	4.05	23.55	8.91	1375.17
Max	24.19	73.82	105.17	14.08	9.02	6.31	28.54	22.49	2936.28
LSD	3.88	6.51	7.39	0.86	1.09	0.69	4.62	3.69	770.43
CV	21.98	7.51	5.93	10.78	13.40	13.25	25.89	24.08	38.50
Heritability (broad)	0.36	0.72	0.64	0.12	0.73	0.74	0.22	0.77	0.54

*, **,*** = Significance at 5, 1 and 0.1%, respectively; ns= not significant PEDLN= Peduncle length (cm). DF50, Days to 50% flowering; MAT90, Days to 90% maturity; SDPPOD, Seeds per pod; SDLEN, Seed length (mm); SDWDT, Seed width (mm); PODSPP, Pods per plant; HSDWT, 100-seed weight (g); GRNYLD, Grain yield (kg/ha); CV, Coefficient of Variation; LSD, Least Significant Difference.

**Figure 1:**
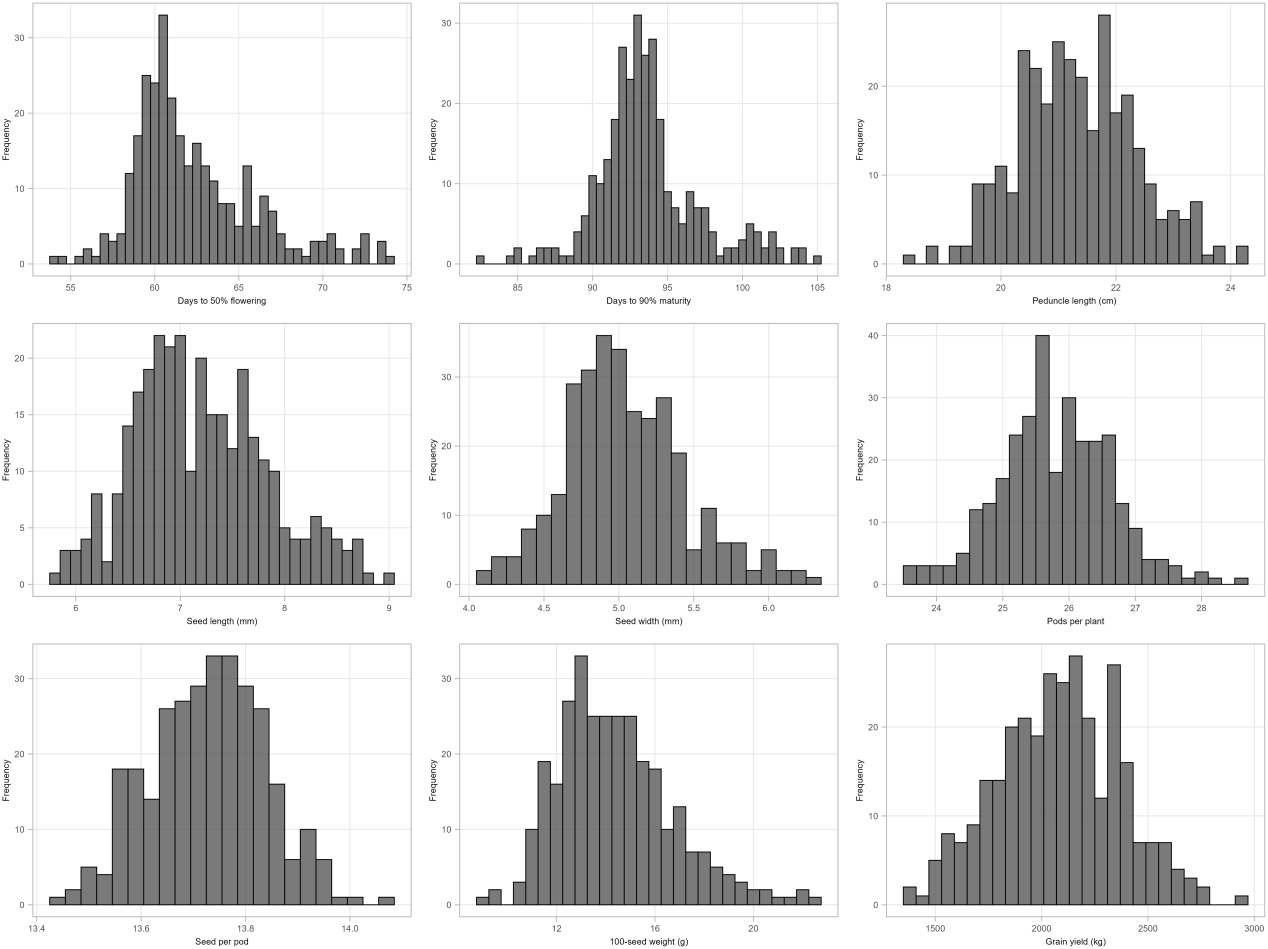
Histogram showing the distribution of morphological traits (BLUPs) measured for 306 cowpea genotypes across two locations: Days to 50% flowering, Days to 90% maturity, Peduncle length (cm), Seed length (mm), Seed width (mm), Pods per plant, Seeds per pod, 100-seed weight and Grain yield.

The mean performance of cowpea genotypes for the traits at each location and across the two locations is presented in **Supplementary Table S3** and the frequency distribution of traits is presented in **Figure 1**. Chitedze had a significantly higher grain yield (2137.24 to 3201.82 kg/ha) compared to Bunda (370.08 to 2813.57 kg/ha). Combined analysis averaged 2080.09 kg/ha. MWcp24 had the highest grain yield (2936.28 kg/ha), while TVu-3101 had the lowest (1375.16 kg/ha). For 100-seed weight, the highest was MWcp03 (22.49 g), and the lowest was TVu-17060 (8.91 g). Flowering and Maturity, TVu-16325 flowered earliest (54.06 days) while Sanzi matured earliest (82.39 days). For seed traits, the mean seed length was 7.20 mm and seed width was 5.04 mm. Pods per plant averaged 25.79, ranging from 23.55 (TVu-1797) to 28.54 (TVu-17526). Peduncle length averaged 21.32 cm, ranging from 18.36 cm (TVu-1797) to 24.19 cm (MWcp27). In terms of variability, the highest coefficient of variation was observed for grain yield (38.5%) and the lowest coefficient of variation was observed for the number of days to 90% maturity (5.9%) and the days to 50% flowering (7.5%).Relationship between grain yield and other traits.

**Figure 2:**
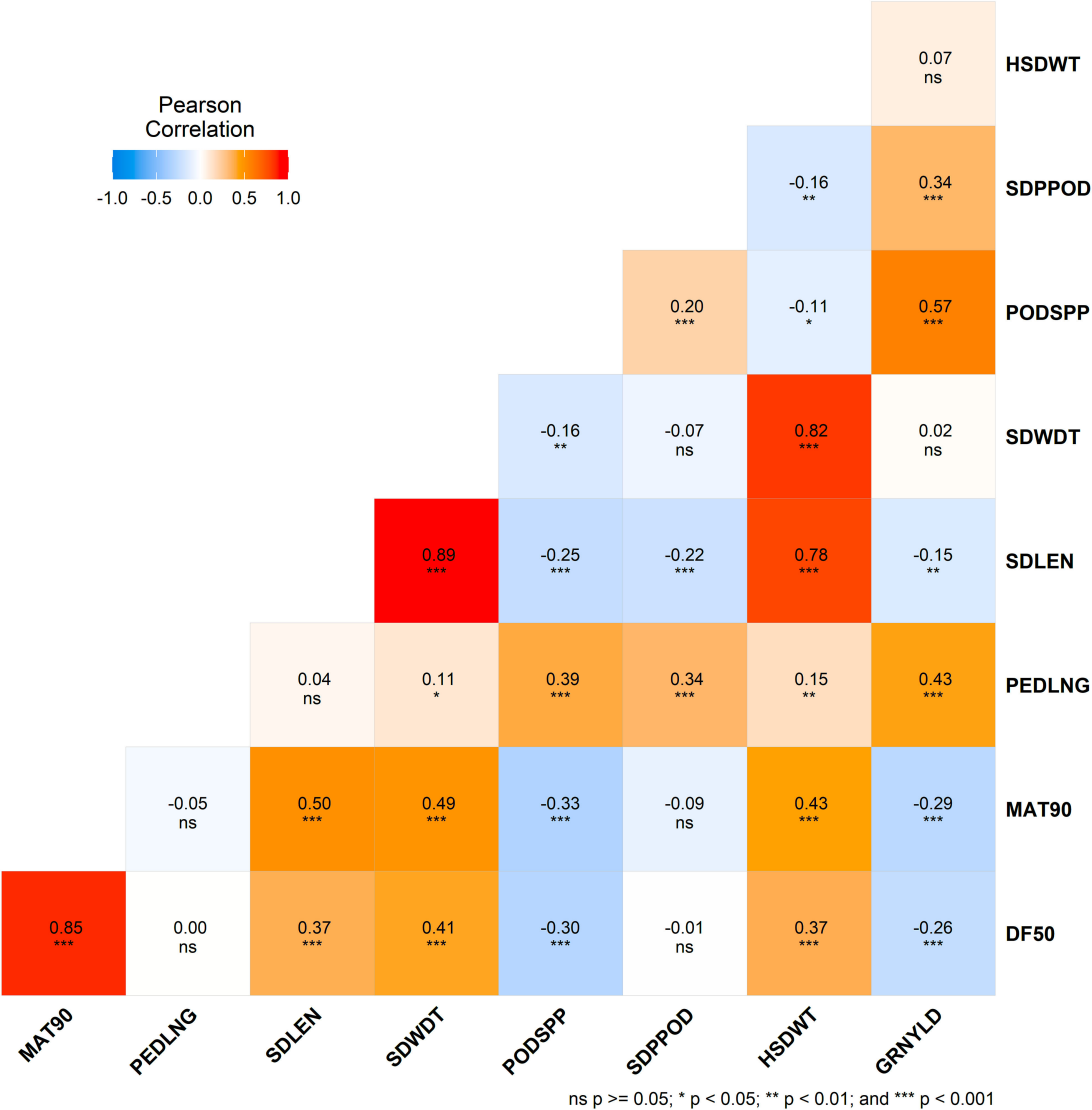
Correlation matrix heatmap showing Pearson's correlation coefficients for morphological traits. Each cell is color-coded based on the correlation value, with red representing positive correlations and blue representing negative correlations. Asterisks in each cell denote the level of significance: ns (not significant), * (p<0.05), ** (p < 0.01), and *** (p < 0.001).

Grain yield was significantly (p < 0.001) positively correlated with peduncle length (r = 0.43), seeds per pod (r = 0.34), and pods per plant (r = 0.57). However, it was negatively correlated (p < 0.001) with days to 50% flowering (r = -0.26) and days to 90% maturity (r = -0.29). Pods per plant was significantly negatively correlated with flowering time, maturity time, and seed length (all p < 0.001). 100-seed weight was also significantly associated with seed length, seed width, flowering time and maturity time (p < 0.001). Positive correlations were also found among other traits in the study (**Figure 2**).

### 3.2 Principal component analysis on phenotypic traits

**Figure 3:**
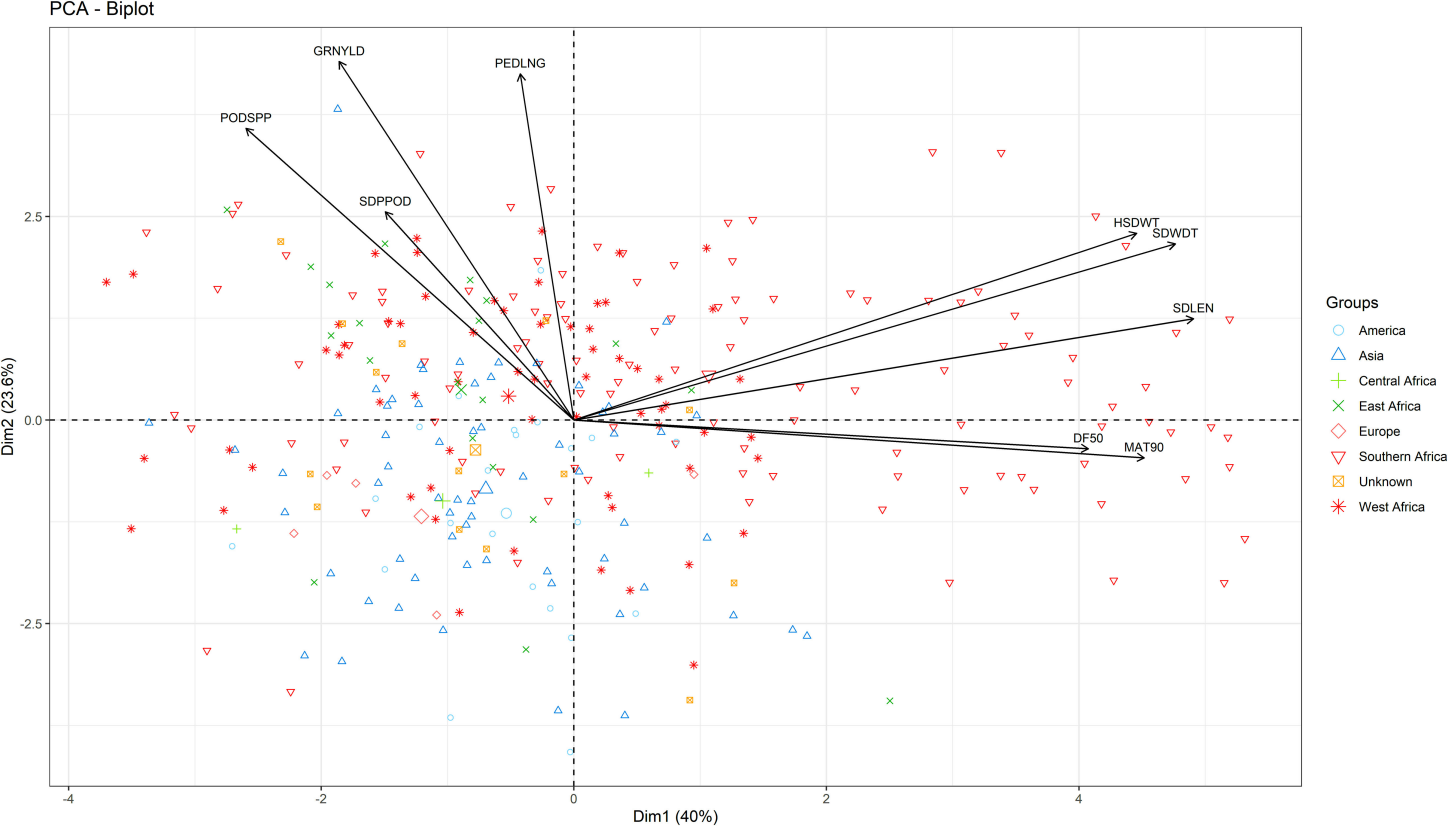
Biplot of the first two dimensions of the principal component analysis (PCA) for the 306 genotypes based on their morphological traits.

Three principal components (PCs) were identified with eigenvalues greater than 1 and collectively accounted for 77% of total variation. PC1 (40% variation) was associated with seed width, seed length, 100-seed weight, days to 90% maturity, and days to 50% flowering (**Figure 3**). PC2 (23.6% variability) was characterized by grain yield, peduncle length, and pod per plant. Lastly, PC3 (13.4% variation) was mostly associated with seeds per pod, days to 50% flowering and days to maturity.

### 3.3 Cowpea genotype clustering based on principal component analysis

**Figure 4:**
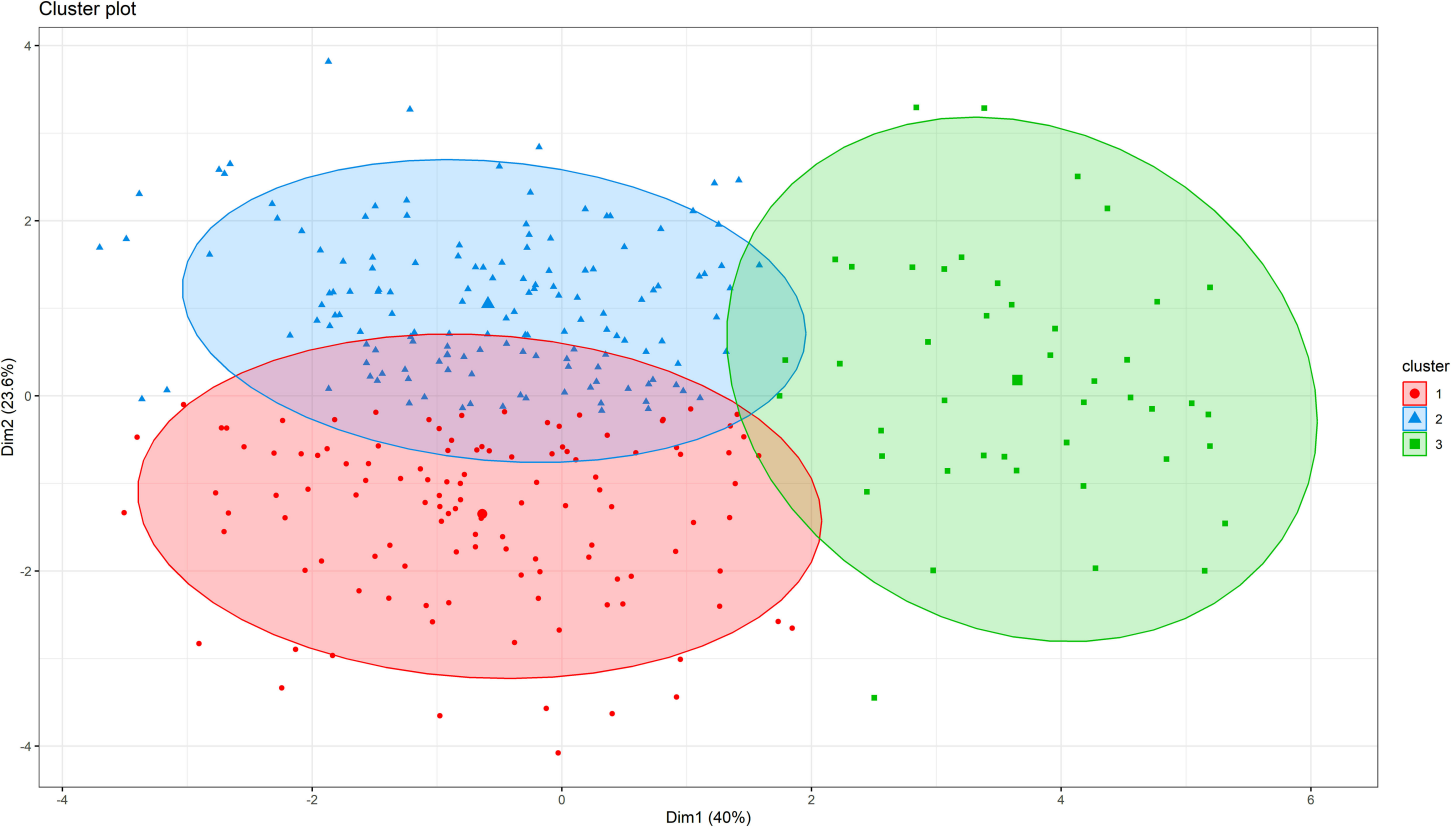
Hierarchical clustering on principal components (HCPC) analysis showing the number of clusters and the distribution of individuals within each cluster for cowpea genotypes.

In hierarchical clustering based on principal component analysis, 306 genotypes were grouped into three distinct clusters (**Figure 4**). The trait mean performances in each cluster are indicated in **Table 2**. Cluster 1 (38.6% of the genotypes) grouped genotypes mainly from Asia (39), West Africa (22), Southern Africa (20), America (17), unknown (8), East Africa (5), Europe (5), and Central Africa (2). These genotypes were characterized by shorter peduncle length, shorter seed length, narrower seed width, and a lower 100-seed weight. Cluster 2 (47.1% of the genotypes), on the other hand, mostly constituted genotypes from Southern Africa (54) and West Africa (42), with others from Asia (23), East Africa (14), unknown (6) and America (5). Typical characteristics for these were longer peduncle length, more pods per plant, and higher grain yield. Lastly, cluster 3 (14.4%) was dominated by genotypes from Southern Africa (43) and one from East Africa (1), and they were characterized by the heaviest 100-seed weight, the longest time to flower, and the longest time to mature.

**Table 2:** Mean and standard deviation of cowpea genotype in clusters.

Trait	Cluster 1 (N=118)	Cluster 2 (N=144)	Cluster 3 (N=44)
Peduncle length (cm)	20.64 ± 0.80	21.91 ± 0.85	21.26 ± 0.97
Days to 50% flowering	61.41 ± 2.79	61.25 ± 2.35	68.73 ± 3.08
Days to 90% maturity	92.74 ± 2.66	92.61 ± 2.25	99.54 ± 3.01
Seeds per pod	13.69 ± 0.10	13.77 ± 0.10	13.69 ± 0.10
Seed length (mm)	6.90 ± 0.54	7.16 ± 0.46	8.15 ± 0.43
Seed width (mm)	4.80 ± 0.30	5.05 ± 0.27	5.67 ± 0.34
Pods per plant	25.39 ± 0.75	26.29 ± 0.69	25.24 ± 0.63
100-seed weight (g)	12.96 ± 1.62	14.44 ± 1.72	17.98 ± 1.99
Grain yield (kg/ha)	1904.53 ± 198.38	2271.71 ± 205.48	1923.78 ± 333.61

### 3.4 Cowpea genetic diversity using SNP markers

A total of 305 cowpea genotypes were evaluated for genetic diversity using 2428 SNP high-quality polymorphic mid-density DArTag SNPs retained after filtering. The SNP variation showed 71.7% transitions and 28.3% transversions, resulting in a transition/transversion ratio of 2.5:1. The frequencies of SNP types A/G, C/T, G/T, A/C, C/G, and A/T were 35.9%, 35.8%, 11.3%, 11.3%, 3.0%, and 2.7%, respectively. The markers had an average call rate of 99% (ranged from 85% to 100%) and a minor allele frequency of 0.27. Summary statistics of diversity indicators are provided in **Supplementary Figure S1**, with additional details in **Table 3**. PIC ranged from 11% to 50%. Across geographic origins, there was an average MAF of 0.31, observed heterozygosity of 0.06, gene diversity of 0.33 and inbreeding coefficient of 0.82. Within geographic origins, the highest mean MAF was observed for Europe (0.36), followed by Central African (0.35), American (0.34), and West African (0.34). Highest observed heterozygosity was observed for Europe and West Africa (0.13 and 0.14, respectively). Mean gene diversity was highest in genotypes with unknown origin (0.42), followed by West Africa (0.40), Europe (0.39), and America (0.38) while lowest in Central Africa (0.25). Europe and West Africa had relatively lower inbreeding coefficients (0.65).

**Table 3:** The genetic diversity statistics of populations based on 2428 single-nucleotide polymorphisms (SNPs).

Population	Countries	N	MAF	Ho	He	Fis
America	USA, Argentina	22	0.34	0.07	0.38	0.82
Asia	India, Philippines	62	0.25	0.07	0.31	0.77
Central Africa	Cameroon	2	0.35	0.00	0.16	0.99
East Africa	Tanzania, Uganda	19	0.22	0.05	0.26	0.82
Europe	Russia, Italy, Hungary	5	0.36	0.13	0.39	0.65
Southern Africa	Malawi, Mozambique, Botswana, South Africa, Zambia, Zimbabwe	117	0.23	0.02	0.30	0.92
Unknown	Unknown	14	0.36	0.02	0.42	0.95
West Africa	Nigeria, Ghana, Benin, Cote d’Ivoire, Senegal	64	0.34	0.14	0.40	0.65
Mean		305	0.31	0.06	0.33	0.82

N, Number of individuals; MAF, Minor allele frequency; Ho, observed heterozygosity; He, Gene diversity; Fis, Inbreeding coefficient.

Pairwise Nei genetic distances revealed varying degrees of genetic differentiation (**Table 4**). The highest genetic distance (0.28) was observed between the Central African and East African genotypes. Other notable distances were between Central Africa and Southern Africa (0.25), Central Africa and Asia (0.25), and Central Africa and Europe (0.23). Minimal distances were observed between West Africa and unknown origins (0.04), America and Europe (0.03), and East Africa and Southern Africa (0.04) populations.

**Table 4:** Pairwise Nei’s genetic distance among cowpea populations.

Population	America	Asia	Central Africa	East Africa	Europe	Southern Africa	Unknown
Asia	0.07						
Central Africa	0.20	0.25					
East Africa	0.11	0.05	0.28				
Europe	0.03	0.09	0.23	0.16			
Southern Africa	0.09	0.04	0.25	0.04	0.13		
Unknown	0.06	0.09	0.19	0.10	0.09	0.09	
West Africa	0.07	0.07	0.22	0.08	0.10	0.06	0.04

**Figure 5:**
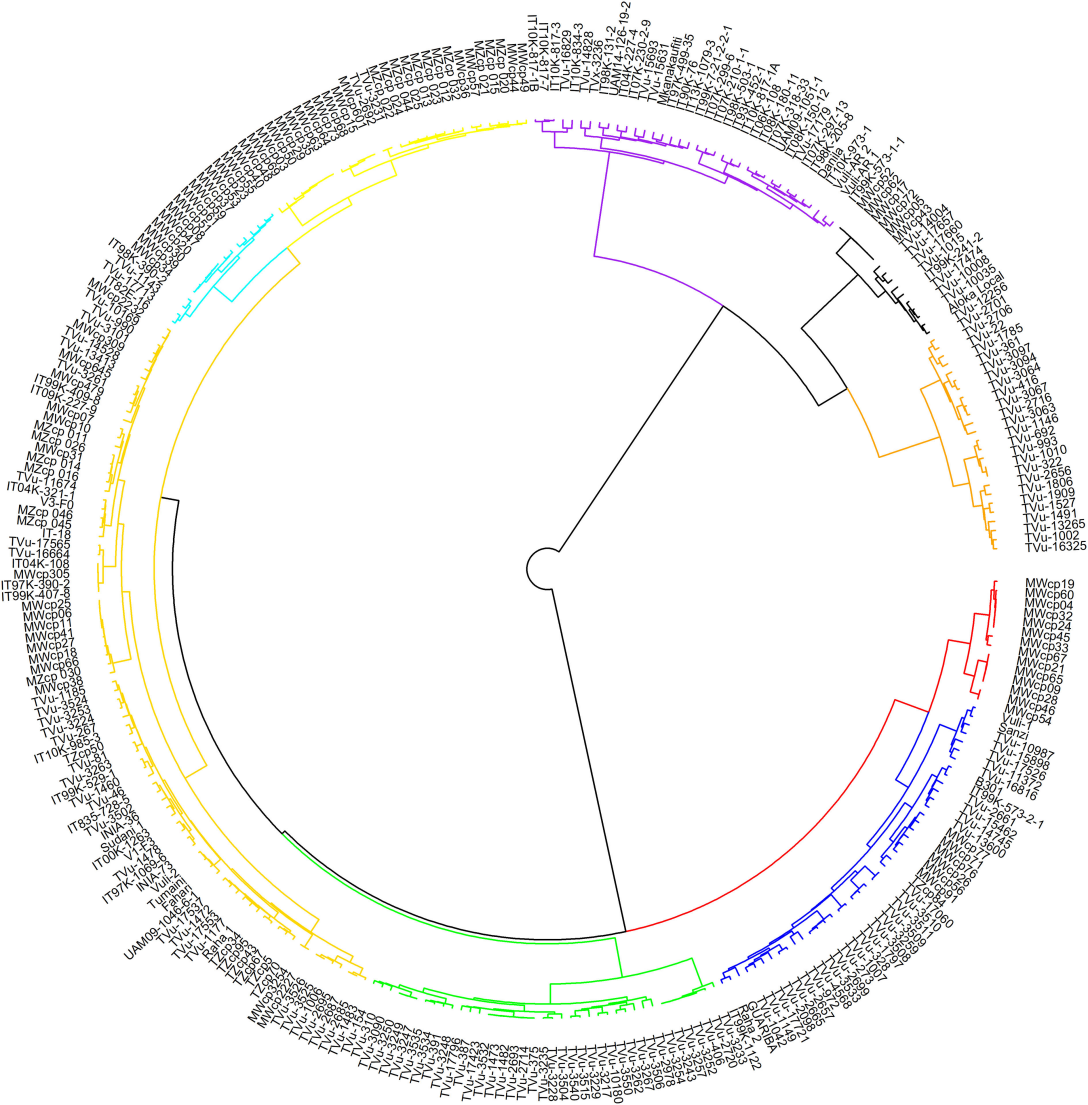
Hierarchical clustering of 305 cowpea genotypes using 2428 SNPs. Different colours represent different clusters.

In hierarchical clustering using the ward.D method (**Figure 5**), cowpea genotypes from the seven geographic origins were grouped into nine clusters. Notable clusters were Cluster 1 with genotypes from Asia, Southern Africa, and East Africa. Cluster 2 is comprised of West Africa and an unknown origin. Cluster 3: Europe and America, and Cluster 4: the Central African genotypes. Cluster 9 consisted entirely of genotypes from Southern Africa, specifically collected from Malawi. The rest of the clusters included genotypes from different regions and countries exhibiting wide variations in distribution.

### 3.5 Population structure and linkage disequilibrium

**Figure 6:**
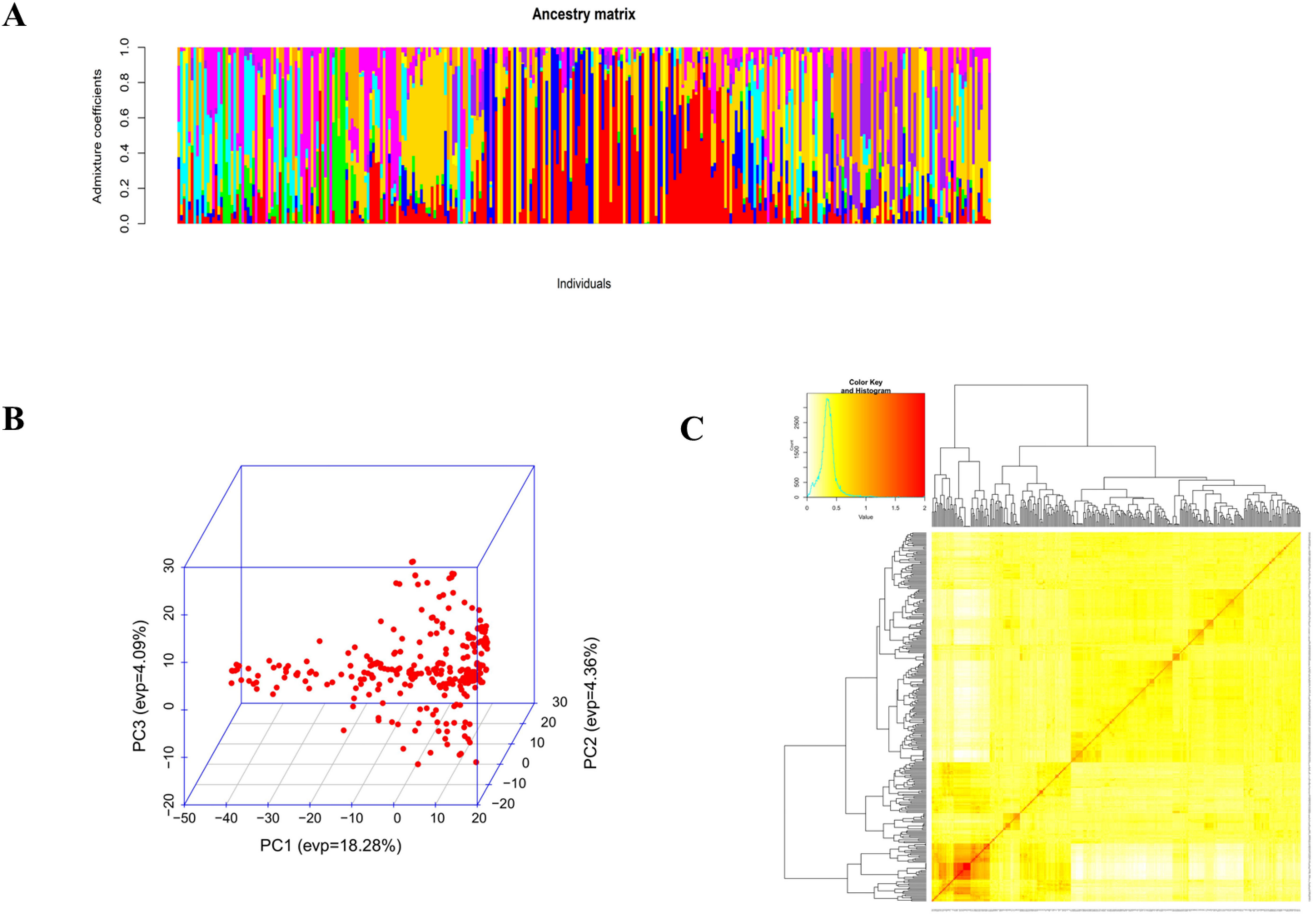
Admixture analysis **(A)**, plot of the first three principal components **(B)**, and heatmap of kinship with the tree **(C)**.

The AMOVA results for cowpea genotypes based on geographic origins showed that 73.1% of the total variation is among individuals within populations (**Table 5**). 15.6% of the total variation is due to differences within individuals, and 9.3% of the total variation is between populations. These three levels contribute to the overall genetic variation. Admixture clustering analysis based on both the cross entropy criterion and the Trancy-widom test (**Supplementary Figure S2**) revealed that the optimal number of genetic clusters in the data, and therefore the number of ancestral populations, was nine (K=9). A barplot using the Q-ancestry matrix (**Figure 6**) revealed that two of the subpopulations had a higher presence of admixture in most genotypes.

**Table 5:** Analysis of molecular variance (AMOVA) showing variation within and between cowpea populations.

Source of variation	DF	SS	MS	Sigma	% Var	Phi	P-value
Between populations	7	49475.1	7067.88	84.72	9.3	0.09	0.01
Within population	297	441477	1486.45	663.4	73.1	0.81	0.01
Within genotypes	305	48696	159.66	159.66	15.6	0.82	0.01
Total	609	539648	886.12	907.78	100		

DF, Degrees of freedom; SS, Sum of squares; MS, Mean square; % Var, Percentage of variation.

Principal component analysis showed that the cumulative percentage contributions of the first two dimensions in the ordination explained 22.6% of the variation. PC1 accounted for 18.28%, and PC2 accounted for 4.36%. Notably, the PCA plot did not reveal a very clear clustering of the genotypes, further suggesting the presence of genetic admixture (**Figure 6**).

**Figure 7:**
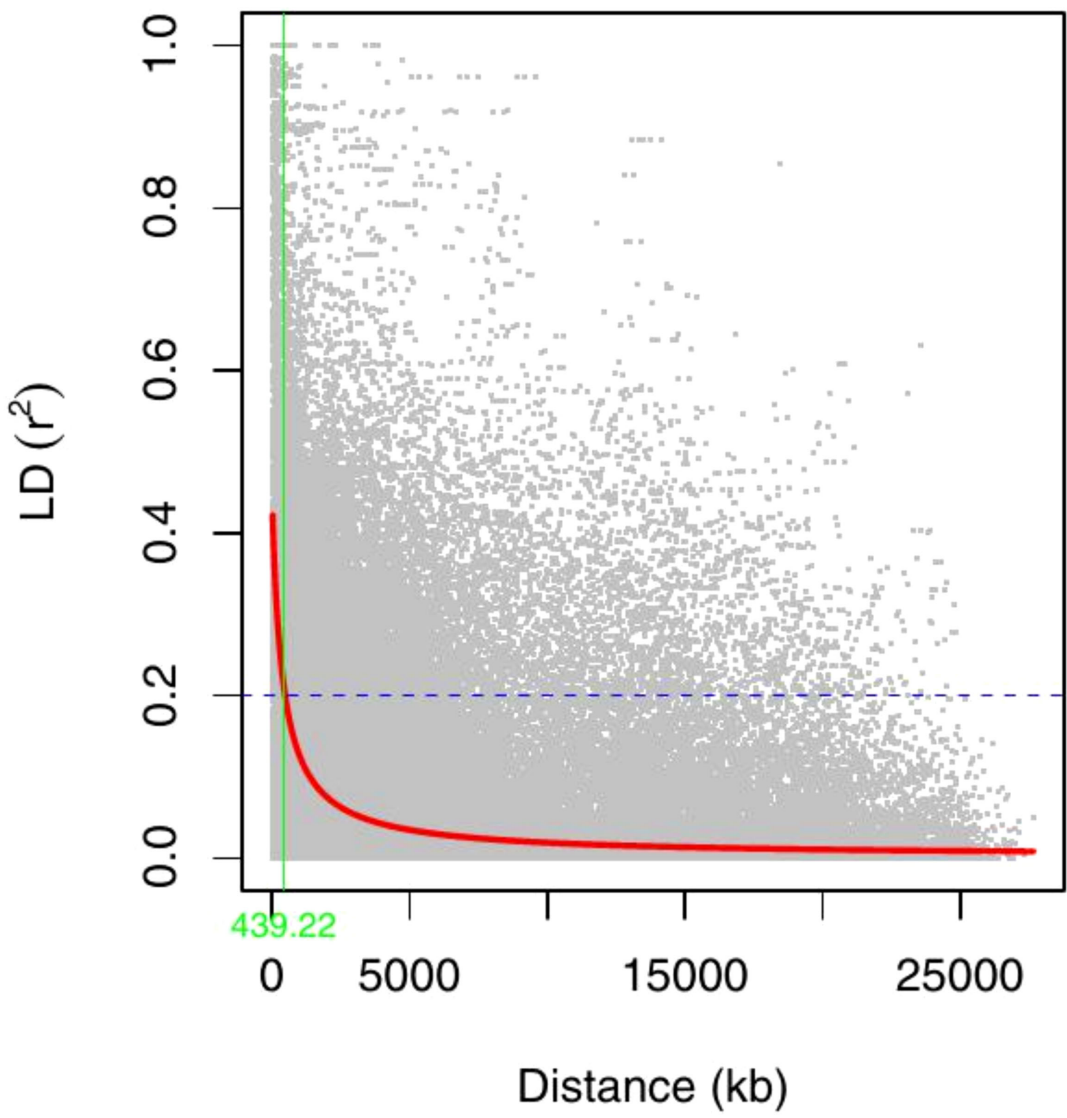
Genome-wide LD decay plot over physical distance based on 2428 SNP markers. The red curve represents the model fits to LD decay. The horizontal magenta dash-line represents the arbitrary threshold for no LD (r^2^ = 0.2). The vertical green line indicates the intersection between the critical r^2^ value and the average map distance (439 kb).

Linkage disequilibrium (LD) decay across all chromosomes was assessed by calculating the squared correlation coefficient (r^2^) for pairs of all SNPs in the 305 cowpea genotypes. A total of 120,125 intra-chromosomal pairs were generated for the LD analysis, with the mean r^2^ of all pairs equal to 0.1. Among these *r* ^2^, 18,260 pairs (15% of the total) exhibited values below the commonly used threshold of 0.2. A non-linear regression model demonstrated a clear decreasing trend in LD as physical distance between SNPs increased (**Figure 7**). Based on this analysis, the average genome-wide LD decay distance was found to be approximately 439 kb.

### 3.6 Genome wide association

**Figure 8:**
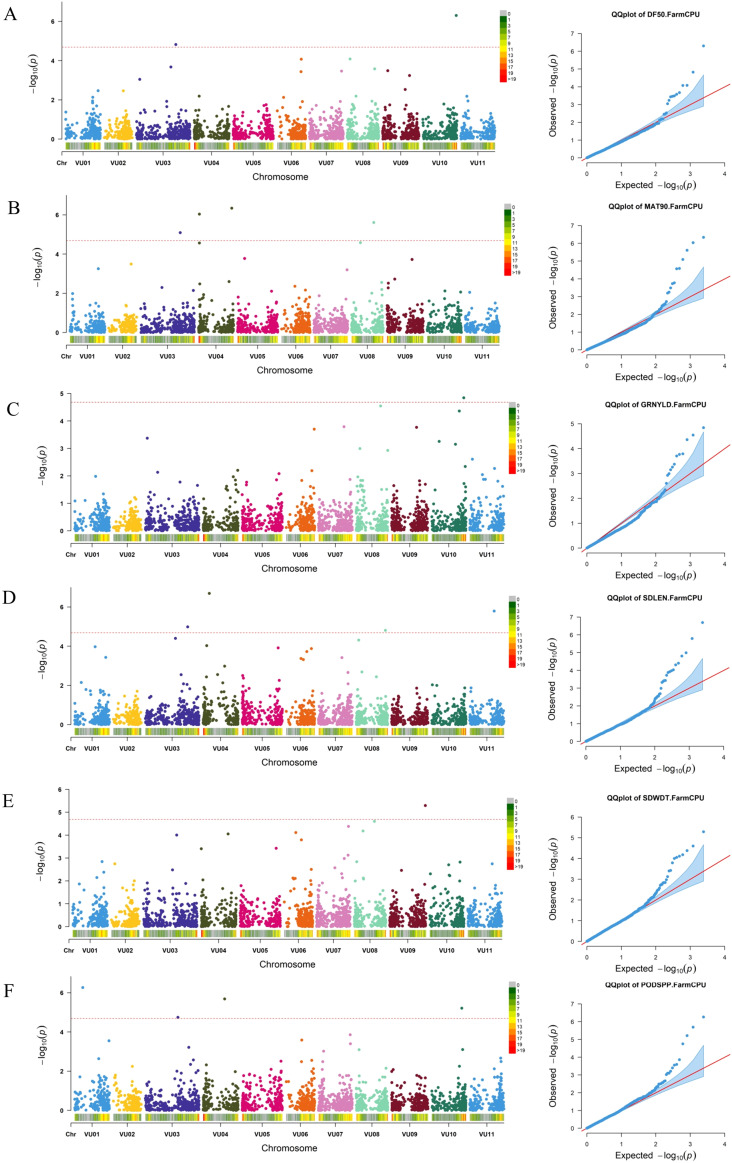
Manhattan and respective-QQ plots for days to 50% flowering **(A)**, days to 90% maturity **(B)**, grain yield **(C)**, seed length **(D)**, seed width **(E)** and pods per plant **(F)** in GWAS panel.

Genome-wide association analysis was conducted using a set of SNPs distributed across all cowpea chromosomes (**Supplementary Figure S3**) and data for nine morphological traits: peduncle length, days to 50% flowering, days to 90% maturity, number of pods per plant, seeds per pod, seed length, seed width, 100-seed weight, and grain yield. The marker-trait associations (MTAs) were declared significant only if their p-values were below the Bonferroni threshold. Based on the Manhattan and Q–Q plots of BLUP values across locations, a total of 16 SNPs were identified as significantly associated with six out of the nine analyzed traits (**Figure 8**; **Supplementary Table S5**). A boxplot of significant MTAs is presented in **Supplementary Figure S4** to visually assess their allelic effects on the phenotype. The highest number of significant SNPs (4) was found on chromosome VU03 and VU04 while no MTAs were identified on chromosome VU02, VU05, and VU07. The number of significant marker-trait associations (MTAs) detected ranged from one MTA for grain yield and seed width to four MTAs for days to 90% maturity, pods per plant, and seed length. Notably, one SNP within the MTAs was associated with multiple traits: SNP 2_49803 on chromosome VU03 (position 48,008,318) was associated with both days to 50% flowering and days to 90% maturity.

Among the identified SNPs, one (2_00383) was significantly associated with grain yield, located on chromosome VU10 at position 38612317. Two SNPs were significantly associated with days to 50% flowering: 2_49803 on chromosome VU03 at position 48,008,318, and 2_28299 on chromosome VU10 at position 40518950. Several SNPs were linked to days to 90% maturity, including 2_49803, 2_18617, 2_50822, and 2_50684, located on chromosomes VU03, VU04, and VU08 at positions 48008318, 1274572, 40470581, and 26860583, respectively.

Seed length was significantly associated with four SNPs distributed across chromosomes VU03, VU04, VU08, and VU11, located at genomic positions 51498849, 7712996, 35389996 and 29429763, respectively. Seed width was significantly associated with one SNP on chromosome VU09 at position 43096254. For pods per plant, four significant SNPs were identified on chromosomes VU01, VU03, VU04, and VU10, located at genomic positions 9793961, 39618312, 26223984, and 36275270, respectively.

## 4 Discussion

The present study characterized a diverse collection of cowpea germplasm originating from different geographic regions. These germplasm resources were assembled to establish a cowpea breeding program in Malawi, with the goal of developing new varieties tailored to demand-driven traits, as highlighted by Chipeta et al. (2024). Given the pressing challenges of food insecurity, economic hardships, and climate change facing the country, the development of demand-driven cowpea varieties represents a crucial strategy to support smallholder farmers and enhance agricultural resilience. Prior to the study, the assembled germplasm remained uncharacterized at both the morphological and molecular levels. However, understanding the genetic diversity and relationships within the cowpea germplasm is crucial for effective breeding and long term conservation (Dagnon et al., 2022). Assessing genetic diversity enables breeding programs to identify potential parental lines for the development improved varieties (Mofokeng et al., 2020; Govindaraj et al., 2015).

The combined analysis of variance revealed highly significant differences for grain yield, 100-seed weight, days to maturity, and other traits, indicating considerable genetic variability present in the germplasm for these traits. This variability is essential for breeding programs as it provides opportunities for selecting and developing superior lines with desirable traits (Gerrano et al., 2015). Additionally, genotypes responded differently in the two locations, which might be attributed to differences in weather, soil fertility, and the genetic makeup of the materials used. Similar findings were reported by Nkhoma et al. (2020). The observed high mean values and variability in the scored traits (grain yield, seed size, and maturity) suggest that ideal parental lines could be identified and utilized for the development of improved varieties. These characterized traits in the study largely align with the preferences of most farmers in Malawi and other countries in East and Southern Africa as previous studies have shown (Chipeta et al., 2024; Hella et al., 2013). The findings thus have a regional impact in terms of usage, given that many countries exhibit similar features in relation to edaphic and climatic conditions.

Improving complex traits, such as grain yield, presents challenges due to their dependence on genetic structures and environmental factors (Walle et al., 2018). To address this, correlation analysis helps assess and identify simple traits directly linked to such complex traits for indirect selection. In the study, grain yield was positively correlated with peduncle length, seeds per pod, and pods per plant. The strong positive correlation suggests that grain yield could be indirectly selected through traits such as the number of seeds per pod. Days to maturity correlated positively with flowering time and seed characteristics but negatively with grain yield and pod count. These results reflect a similar trend observed elsewhere (Meena et al., 2015; Owusu et al., 2021; Walle et al., 2018). The negative correlation between days to maturity and grain yield may be attributed to breeding efforts in cowpea, which have historically focused on improving yield while reducing the growth cycle. In contrast, this study included landraces, which typically have longer growth durations but lower yields, contributing to the observed negative relationship.

Hierarchical clustering based on morphological traits revealed three genetic clusters although geographic origin did not directly influence clustering. Similar findings were reported in Togo and Uganda (Edema et al., 2023; Gbedevi et al., 2021). Cowpea genotypes from the same region exhibit diverse clustering due to factors like germplasm exchange, genetic drift, and environmental variation (Walle et al., 2019). Similarly, the presence of regional and cross-border trade in East and Southern Africa, as well as West and Central Africa could also account for the observed grouping pattern.

In our study, molecular diversity and population structure were evaluated using 2428 markers and 305 cowpea genotypes retained after filtering. The higher frequences of A/G and C/T variants observed align with earlier studies (Abdoulaye et al., 2024; Gbedevi et al., 2021; Xiong et al., 2016).

The polymorphism information content (PIC) value (marker informativeness) for the DarTag markers was 0.38 suggesting that the markers were sufficiently informative (Seo et al., 2020). The minor allele frequency (MAF) was moderate at 0.34. MAF serves to distinguish common and rare variants within the population (Dussault and Boulding, 2018).

Additionally, the average observed heterozygosity (Ho) and gene diversity (He) for the 305 cowpea genotypes were 0.06 and 0.33, respectively. These values are consistent with those reported by Gbedevi et al. (2021) for 70 cowpea accessions (Ho = 0.05, He = 0.31) and by Xiong et al. (2016) for 768 global cowpea germplasm collections (Ho = 0.06, He = 0.35). Although He was moderately low in our study, it remained higher than Ho across all geographic origins. Fatokun et al. (2018) and Gumede et al. (2022) also observed a similar trend while studying the diversity of cowpea accessions using single nucleotide polymorphism.

A higher inbreeding coefficient was observed, with a mean of 0.82 in cowpea populations. This high value is consistent with the crop’s self-pollination nature and low outcrossing rate. Seo et al. (2020) also reported similar findings, noting relatively high Fis values of 0.993 and 0.988 for 376 global and 229 Korean cowpea accessions, respectively. Europe and West Africa had relatively lower inbreeding coefficients (0.65), suggesting less genetic traits being inherited due to relatedness in these regions compared to others. This could potentially indicate a greater genetic diversity within these populations.

These results provide valuable insights into the genetic diversity and structure of cowpea genotypes across different geographical regions, which can be crucial for effective conservation and breeding strategies. Padulosi and Ng (1997) identified West and Central Africa as the hubs of diversity. These findings corroborate this, as West Africa.

The Nei genetic distances further demonstrated that genotypes from all populations (Asia, East Africa, West Africa, Southern Africa, Europe, America, and unknown) exhibited minimal genetic variation among populations, with the exception of those from Central Africa. Gene flow between populations likely contributes to higher genetic variance within populations.

To infer population structure, we conducted an analysis of molecular variance (AMOVA) which revealed that most genetic variation occurs within individuals within the seven populations (73.1% of total variation), with only 9.3% attributed to differences between populations. Similar findings were reported by Abdoulaye et al. (2024); Ongom et al. (2024); Gbedevi et al. (2021); Sarr et al. (2021); Fatokun et al. (2018) and Chen et al. (2017) who reported highest genetic variation between individuals within population. Consequently, the majority of the genetic variation observed in cowpea is attributed to individual variation within population rather than geographical distribution. Admixture analysis and hierarchical clustering on molecular data identified nine main ancestral populations and clusters, respectively, with significant genetic admixture, regardless of geographic origin. The findings indicated that genotypes did not cluster precisely based on their geographical origin. The fact that some genotypes from diverse geographical origins were grouped into the same clusters could imply a certain degree of relatedness among them. This could be due to the transfer of germplasm from one location to another, either through germplasm exchange or cross-border trade. On the other hand, the fact that some genotypes from the same geographical origin were clustered into different groups may suggest some level of genetic differentiation among the populations, as evidenced by high levels of admixture within the population. This phenomenon, where subpopulations are grouped irrespective of geographical origins, has been observed in other studies on genetic diversity in cowpea accessions (Abdoulaye et al., 2024; Gumede et al., 2022). Understanding this structure can aid in selecting breeding parents and optimizing gains from selection.

Determining the genetic basis of complex traits has become increasingly effective with the advancements in genome sequencing (Lonardi et al., 2019) and high-density genotyping platforms (Muñoz-Amatriaín et al., 2017). In this study, a mid-density SNP genotyping panel (Ongom et al., 2024) was used to perform GWAS, a powerful tool for exploring genetic diversity. The association analysis used the FarmCPU algorithm (Liu et al., 2016) implemented through the rMVP package (Yin et al., 2021). FarmCPU integrates fixed effect models (FEM) for marker tests with associated covariates while optimizing these covariates in random effect models (REM), thereby improving power and reducing false positives (Liu et al., 2016). The GWAS identified significant MTAs with six morphological traits for 305 cowpea genotypes including days to 50% flowering, days to 90% maturity, grain yield, seed length, seed width and pods per plant.

Flowering time is an important phenological trait in plant adaptation influencing agronomic traits such as plant growth, plant height, and grain quality (González et al., 2016). Early flowering can be transferred to cultivated cowpea through hybridization with early flowering accessions (Paudel et al., 2021). Early flowering has significant implications for climate resilience and early income generation, especially for smallholder farmers in Malawi. In contrast, late flowering in cowpea is particularly very important for farmers who prioritize vegetable consumption (Chipeta et al., 2024). Two SNPs, located on chromosomes VU03 and VU10, were significantly associated with flowering time, while four SNPs were linked to maturity time. SNP 2_49803 (on VU03) was notable for its positive effect on both traits, indicating proximity to a regulatory factor influencing both flowering and maturity. Paudel et al. (2021) identified seven significant SNPs associated with flowering time in cowpea linked to candidate genes such as FT, GI, CRY2, LSH3, UGT87A2, LIF2, and HTA9. The differences in SNP associations between studies suggest that flowering time may be governed by multiple loci influenced by factors like marker density, population structure, and environmental conditions, necessitating further validation.

Four SNPs significantly associated with days to 50% flowering, days to 90% maturity, seed length, and the number of pods per plant were identified on chromosome VU03. Additionally, four SNPs significantly associated with three traits (days to 90% maturity, seed length, and the number of pods per plant) were identified on chromosome VU04. These results suggest that these chromosomes play a crucial role in cowpea for multiple trait selection.

Seed size, which varies among genotypes, is one of the key yield determinants with its genetic basis not yet well understood (Lo et al., 2019). Four significant SNPs were identified on chromosomes VU03, VU04, VU08, and VU11, each associated with longer seed size. These findings underscore the importance of these alleles for improving marketability and meeting consumer demands in Malawi. However, seed width was associated with a single SNP (2_22597) on chromosome VU09. The study also identified four SNPs associated with number of pods per plant located on chromosomes VU01, VU03, VU04, and VU10. Pods per plant directly influence grain yield potential in cowpea, making these SNPs valuable for breeding programs aimed at developing high yielding varieties. Given the complexity of grain yield as a trait (Walle et al., 2018), the association between grain yield and a SNP on chromosome VU10 (position 38,612,317) with negative effect highlight an undesirable allele that should be avoided in breeding programs focused on yield improvement.

There are only a few published GWAS reports on grain yield and morphological traits in cowpea and most of these predate the availability of a completely annotated cowpea genome (Wu et al., 2024). Our findings provide a foundation for several downstream analysis and applications including candidate gene screening, functional validation of identified QTLs, and integration into marker-assisted selection, particularly in Malawi, where the technique is still in its early stages. While the findings are promising, further validation of the identified MTAs is necessary. Future candidate gene screening could also deepen our understanding of the genetic architecture of these traits, increasing the efficiency of breeding programs by allowing for the genetic selection of desirable traits and decreasing the need for extensive phenotyping which is costly.

The observed genetic variations in this research could play a crucial role in cowpea breeding programs in Malawi. These differences are a beneficial genetic asset for the selection of parent lines for the development of more productive cowpea varieties. The diversity among populations implies that these groups could serve as vital germplasm sources for breeding initiatives with a restricted germplasm pool. Conversely, the diversity seen within individuals shows that we currently possess a varied germplasm pool that can be employed for the creation of different varieties. If this germplasm pool is conserved and used wisely, it could result in a substantial increase in genetic recombination among offspring, potentially amplifying the genetic advantages derived from crop selection.

## 5 Conclusion

The study identified significant genetic diversity among cowpea genotypes based on both morphological traits and DArTag SNP markers. Variations were observed in grain yield, 100-seed weight, number of seeds per pod, number of pods per plant, and days to 90% maturity, indicating room for improvement. Significant MTAs associated with some of the traits were also identified. Additionally, insights into population structure within the genotypes can guide the selection of parental lines to enhance selection gains. The study’s findings underscore the relevance of both morphological and molecular characterization methods. Overall, this research serves as a valuable resource for selecting desirable cowpea accessions in breeding programs and contributes to improving cowpea breeding efforts in Malawi.

## Data Availability

The datasets presented in this study can be found in online repositories. The names of the repository/repositories and accession number(s) can be found in the article/**Supplementary Material**.

## References

[B1] Abberton M. Sansa O. Ariyo J. Paliwal R. Ige A. Dieng I.. (2024). Phenotypic data of 112 cowpea accessions evaluated under well-watered and water-stressed conditions across 3 locations 2020, 2021 & 2022. Int. Institute Trop. Agric. doi: 10.25502/15QT-7Z62/D

[B2] Abdoulaye A. K. Wireko A. K. Annor B. Adejumobi I. I. Maina F. Maazou A. R. S.. (2024). DArTseq-based genome-wide SNP markers reveal limited genetic diversity and highly structured population in assembled West African cowpea germplasm. Sci. Afr., e02065. doi: 10.1016/j.sciaf.2024.e02065

[B3] Alemu M. Asfaw Z. Woldu Z. Fenta B. A. Medvecky B. (2016). Cowpea (Vigna unguiculata (L.) Walp.) (Fabaceae) landrace diversity in Northern Ethiopia. Int. J. Biodiversity Conserv. 8, 297–309. doi: 10.5897/IJBC2016.0946

[B4] Alvarado G. Rodríguez F. M. Pacheco A. Burgueño J. Crossa J. Vargas M.. (2020). META-R: A software to analyze data from multi-environment plant breeding trials. Crop J. 8, 745–756. doi: 10.1016/j.cj.2020.03.010

[B5] Badiane F. A. Diouf M. Diouf. D. (2014). “Cowpea,” in Broadening the Genetic Base of Grain Legumes. Eds. Singh M. Bisht I. Dutta M. (Springer, New Delhi).

[B6] Bates D. Maechler M. Bolker B. Walker S. Christensen R. H. B. Singmann H.., (2015). Package ‘lme4’. convergence. 12, 2.

[B7] Boukar O. Belko N. Chamarthi S. Togola A. Batieno J. Owusu E.. (2018). Cowpea (Vigna unguiculata): Genetics, genomics and breeding. Plant Breed. 138, 415–424. doi: 10.1111/pbr.12589

[B8] Boukar O. Belko N. Chamarthi S. Togola A. Batieno J. Owusu E.. (2019). Cowpea (Vigna unguiculata): genetics, genomics and breeding. Plant Breed. 138, 415–424. doi: 10.1111/pbr.12589

[B9] Boukar O. Fatokun C. A. Huynh B. L. Roberts P. A. Close. T. J. (2016). Genomic tools in cowpea breeding programs: status and perspectives. Front. Plant Sci. 7, 757. doi: 10.3389/fpls.2016.00757 27375632 PMC4891349

[B10] Boukar O. Massawe F. Muranaka S. Franco J. Maziya-Dixon B. Singh B.. (2011). Evaluation of cowpea germplasm lines for protein and mineral concentrations in grains. Plant Genet. Resour. 9, 515–522. doi: 10.1017/S1479262111000815

[B11] Bradbury P. J. Zhang Z. Kroon D. E. Casstevens T. M. Ramdoss Y. Buckler E. S. (2007). TASSEL: software for association mapping of complex traits in diverse samples. Bioinformatics 23, 2633–2635. doi: 10.1093/bioinformatics/btm308 17586829

[B12] Chang C. C. Chow C. C. Tellier L. C. Vattikuti S. Purcell S. M. Lee. (2015). Second-generation PLINK: rising to the challenge of larger and richer datasets. Gigascience 4, s13742–s13015. doi: 10.1186/s13742-015-0047-8 PMC4342193 25722852

[B13] Chen H. Chen H. Hu L. Wang L. Wang S. Wang M. L.. (2017). Genetic diversity and a population structure analysis of accessions in the Chinese cowpea [Vigna unguiculata (L.) Walp.] germplasm collection. Crop J. 5, 363–372. doi: 10.1016/j.cj.2017.04.002

[B14] Chipeta M. M. Kampanje-Phiri J. Moyo D. Colial H. Tamba M. Belarmino D.. (2024). Understanding specific gender dynamics in the cowpea value chain for key traits to inform cowpea breeding programs in Malawi, Mozambique and Tanzania. Front. Sociology 9. doi: 10.3389/fsoc.2024.1254292 PMC10901980 38425671

[B15] Coulibaly O. Alene A. D. Abdoulaye T. Chianu C. Manyong V. Aitchedji C.. (2010). Baseline assessment of cowpea breeding and seed delivery efforts to enhance poverty impacts in sub-Saharan Africa. (Telengana, India: ICRISAT). Available online at: https://www.Icrisat.Org/What-We-Do/Impi/Projects/Tl2-Publications/Research-Reports/Rr-Cwpsbean.PdfCRP.

[B16] D’Andrea A. C. Kahlheber S. Logan A. L. Watson. D. J. (2007). Early domesticated cowpea (Vigna unguiculata) from Central Ghana. Antiquity 81, 686–698. doi: 10.1017/S0003598X00095661

[B17] Dagnon Y. D. Palanga K. K. Bammite D. Bodian A. Akabassi G. C. Foncéka D.. (2022). Genetic diversity and population structure of cowpea [Vigna unguiculata (L.) Walp.] accessions from Togo using SSR markers. PloS One 17, e0252362. doi: 10.1371/journal.pone.0252362 36197899 PMC9534403

[B18] De Vicente M. C. Fulton T. (2003). Using molecular marker technology in studies on plant genetic diversity. Illus. Nelly Giraldo (Ithaca, New York, USA: IPGRI, Rome, Italy and Institute for Genetic Diversity).

[B19] Dussault F. M. Boulding E. G. (2018). Effect of minor allele frequency on the number of single nucleotide polymorphisms needed for accurate parentage assignment: A methodology illustrated using Atlantic salmon. Aquaculture Res. 49, 1368–1372. doi: 10.1111/are.13566

[B20] Edema R. Adjei E. A. Ozimati A. A. Tusiime S. M. Badji A. Ibanda A.. (2023). Genetic diversity of cowpea parental lines assembled for breeding in Uganda. Plant Mol. Biol. Rep. 41, 713–725. doi: 10.1007/s11105-023-01394-6

[B21] FAOSTAT (2024). Food and Agriculture Organization Corporate Statistical Database. Available online at: http://www.fao.org/faostat/en/data/QC (Accessed November 5, 2024).

[B22] Fatokun C. Girma G. Abberton M. Gedil M. Unachukwu N. Oyatomi O.. (2018). Genetic diversity and population structure of a mini-core subset from the world cowpea (Vigna unguiculata (L.) Walp.) germplasm collection. Sci. Rep. 8, 16035. doi: 10.1038/s41598-018-34555-9 30375510 PMC6207765

[B23] Frichot E. François O. (2015). LEA: An R package for landscape and ecological association studies. Methods Ecol. Evol. 6, 925–929. doi: 10.1111/mee3.2015.6.issue-8

[B24] Gbedevi K. M. Boukar O. Ishikawa H. Abe A. Ongom P. O. Unachukwu N.. (2021). Genetic diversity and population structure of cowpea [Vigna unguiculata (L.) walp.] germplasm collected from Togo based on DArT markers. Genes 12, 9. doi: 10.3390/genes12091451 PMC8465771 34573433

[B25] Gerrano A. Adebola P. Jansen van Rensburg W. Venter S. (2015). Genetic variability and heritability estimates of nutritional composition in the leaves of selected cowpea genotypes [Vigna unguiculata (L.) walp. HortScience: A Publ. Am. Soc. Hortic. Sci. 50, 1435–1440. doi: 10.21273/HORTSCI.50.10.1435

[B26] Gomes A. M. F. Nhantumbo N. Ferreira-Pinto M. Massinga R. Ramalho J. C. Ribeiro-Barros A. I. (2019). “Breeding elite cowpea [Vigna unguiculata (L.) walp] varieties for improved food security and income in Africa: opportunities and challenges,” in Legume Crops. Ed. El-Esawi M. A. (London, United Kingdom: Intech), 14. doi: 10.5772/intechopen.84985

[B27] González A. M. Yuste-Lisbona F. J. Saburido S. Bretones S. De Ron A. M. Lozano R.. (2016). Major contribution of flowering time and vegetative growth to plant production in common bean as deduced from a comparative genetic mapping. Front. Plant Sci. 7, 1940. doi: 10.3389/fpls.2016.01940 28082996 PMC5183638

[B28] Govindaraj M. Vetriventhan M. Srinivasan M. (2015). Importance of genetic diversity assessment in crop plants and its recent advances: An overview of its analytical perspectives. Genet. Res. Int. 2015, 431487. doi: 10.1155/2015/431487 25874132 PMC4383386

[B29] Gruber B. Unmack P. J. Berry O. F. Georges A. (2018). dartr: An r package to facilitate analysis of SNP data generated from reduced representation genome sequencing. Mol. Ecol. Resour. 18, 691–699. doi: 10.1111/men.2018.18.issue-3 29266847

[B30] Gu Z. Gu M. Z. GlobalOptions I. (2024). Package ‘circlize.’.

[B31] Guimarães J. B. Nunes C. Pereira G. Gomes A. Nhantumbo N. Cabrita P.. (2023). Genetic diversity and population structure of cowpea (Vigna unguiculata (L.) walp.) landraces from Portugal and Mozambique. Plants (Basel Switzerland) 12, 846. doi: 10.3390/plants12040846 36840194 PMC9963184

[B32] Gumede M. T. Gerrano A. S. Amelework A. B. Modi A. T. (2022). Analysis of genetic diversity and population structure of cowpea (Vigna unguiculata (L.) walp) genotypes using single nucleotide polymorphism markers. Plants (Basel Switzerland) 11, 3480. doi: 10.3390/plants11243480 36559592 PMC9780845

[B33] Hella J. P. Chilongo T. Mbwag A. M. Bokosi J. Kabambe V. Riches C.. (2013). Participatory market led cowpea breeding in sub Saharan Africa: Evidence pathway from Malawi and Tanzania. Merit. Res. J. Agric. Sci. Soil Sci. 1, 011–018. Available online at: http://www.suaire.sua.ac.tz/handle/123456789/3765.

[B34] International Board for Plant Genetic Resources (1983). Cowpea descriptors (Rome, Italy: Food and Agriculture Organization of the United Nations).

[B35] International Institute of Tropical Agriculture (IITA) (2016). Available online at: https://www.iita.org/cropsnew/cowpea/1620977076200-deb9bc13-b09b (Accessed 5/07/2024).

[B36] Intertek-Agritech (2016). Agri-services CGIAR HTPG PROJECT sampling instructions for SNP verification and routine SNP analysis. (Excellence in Breeding (EiB) platform). Available online at: https://dev.excellenceinbreeding.org/sites/default/files/manual/Sampling%20instructions%20CGIAR%20HTPG%20Project_0.pdf (Accessed November 15, 2021)

[B37] Jombart T. (2008). adegenet: a R package for the multivariate analysis of genetic markers. Bioinformatics 24, 1403–1405. doi: 10.1093/bioinformatics/btn129 18397895

[B38] Kamvar Z. N. Tabima J. F. Grünwald N. J. (2014). Poppr: an R package for genetic analysis of populations with clonal, partially clonal, and/or sexual reproduction. PeerJ. 2, e281. doi: 10.7717/peerj.281 24688859 PMC3961149

[B39] Kassambara A. Mundt F. (2017). Package ‘factoextra.’ Extract and visualize the results of multivariate data analyses 76.

[B40] Ketema S. Tesfaye B. Keneni G. Fenta B. A. Assefa E. Greliche N.. (2020). DArTSeq SNP-based markers revealed high genetic diversity and structured population in Ethiopian cowpea [Vigna unguiculata (L.) Walp] germplasms. PloS One 15, e0239122. doi: 10.1371/journal.pone.0239122 33031381 PMC7544073

[B41] Kouam E. B. Pasquet R. S. Campagne P. Tignegre J. B. Thoen K. Gaudin R.. (2012). Genetic structure and mating system of wild cowpea populations in West Africa. BMC Plant Biol. 12, 113. doi: 10.1186/1471-2229-12-113 22827925 PMC3438136

[B42] Koura A. A. Kena A. W. Annor B. Adejumobi I. I. Maina F. Maazou A. R. S.. (2024). DArTseq-based genome-wide SNP markers reveal limited genetic diversity and highly structured population in assembled West African cowpea germplasm. Scientific African. 23, e02065. doi: 10.1016/j.sciaf.2024.e02065

[B43] Lê S. Josse J. Husson F. (2008). FactoMineR: an R package for multivariate analysis. J. Stat. Software 25, 1–18. doi: 10.18637/jss.v025.i01

[B44] Liu X. Huang M. Fan B. Buckler E. S. Zhang Z. (2016). Iterative usage of fixed and random effect models for powerful and efficient genome-wide association studies. PloS Genet. 12, e1005767. doi: 10.1371/journal.pgen.1005767 26828793 PMC4734661

[B45] Lo S. Muñoz-Amatriaín M. Hokin S. A. Cisse N. Roberts P. A. Farmer A. D.. (2019). A genome-wide association and meta-analysis reveal regions associated with seed size in cowpea [Vigna unguiculata (L.) Walp. Theor. Appl. Genet. 132, 3079–3087. doi: 10.1007/s00122-019-03407-z 31367839 PMC6791911

[B46] Lonardi S. Muñoz-Amatriain M. Liang Q. Shu S. Wanamaker S. Lo S. L.. (2019). The genome of cowpea (Vigna unguiculata [L.] Walp.). Plant journal: Cell Mol. Biol. 98, 767–782. doi: 10.1111/tpj.14349 PMC6852540 31017340

[B47] Mackay I. Powell W. (2007). Methods for linkage disequilibrium mapping in crops. Trends Plant Sci. 12, 57–63. doi: 10.1016/j.tplants.2006.12.001 17224302

[B48] Mafakheri K. Bihamta M. R. Abbasi A. R. (2017). Assessment of genetic diversity in cowpea (Vigna unguiculata L.) germplasm using morphological and molecular characterisation. Cogent Food Agric. 3, 1327092. doi: 10.1080/23311932.2017.1327092

[B49] Meena H. Krishna K. R. Singh B. (2015). Character associations between seed yield and its components traits in cowpea [Vigna unguiculata (L.) Walp]. Indian J. Agric. Res. 49, 567–570. doi: 10.18805/ijare.v49i6.6688

[B50] Mofokeng M. A. Mashilo J. Rantso P. Shimelis H. (2020). Genetic variation and genetic advance in cowpea based on yield and yield-related traits. Acta Agriculturae Scandinavica Section B-Soil Plant Sci. 70, 381–391. doi: 10.1080/09064710.2020.1749295

[B51] Muñoz-Amatriaín M. Mirebrahim H. Xu P. Wanamaker S. I. Luo M. Alhakami H.. (2017). Genome resources for climate-resilient cowpea, an essential crop for food security. Plant J. 89, 1042–1054. doi: 10.1111/tpj.13404 27775877

[B52] Muranaka S. Shono M. Myoda T. Takeuchi J. Franco J. Nakazawa Y.. (2015). Genetic diversity of physical, nutritional and functional properties of cowpea grain and relationships among the traits. Plant Genet. Resour. 1, 1–10. doi: 10.1017/S147926211500009X

[B53] Nkhoma N. Shimelis H. Laing M. D. Shayanowako A. Mathew I. (2020). Assessing the genetic diversity of cowpea [Vigna unguiculata (L.) Walp.] germplasm collections using phenotypic traits and SNP markers. BMC Genet. 21, 110. doi: 10.1186/s12863-020-00914-7 32948123 PMC7501654

[B54] Nkongolo K. Bokosi J. Malusi M. Vokhiwa Z. Mphepo M. (2009). Agronomic, culinary, and genetic characterization of selected cowpea elite lines using farmers’ and breeder’s knowledge: A case study from Malawi. Afr. J. Plant Sci. 3, 147–156.

[B55] Olivoto T. Lúcio A. D. C. (2020). metan: An R package for multi-environment trial analysis. Methods Ecol. Evol. 11, 783–789. doi: 10.1111/2041-210X.13384

[B56] Omomowo O. I. Babalola O. O. (2021). Constraints and prospects of improving cowpea productivity to ensure food, nutritional security and environmental sustainability. Front. Plant Sci. 12. doi: 10.3389/fpls.2021.751731 PMC8570086 34745184

[B57] Ongom P. O. Fatokun C. Togola A. Garcia Oliveira A. L. Ng E. H. Kilian A.. (2024). A mid-density single-nucleotide polymorphism panel for molecular applications in cowpea (*Vigna unguiculata* (L.) Walp). Int. J. Genomics 2024, 9912987. doi: 10.1155/2024/9912987 38235497 PMC10791481

[B58] Owusu E. Y. Karikari B. Kusi F. Haruna M. Amoah R. A. Attamah P.. (2021). Genetic variability, heritability and correlation analysis among maturity and yield traits in Cowpea (Vigna unguiculata (L) Walp) in Northern Ghana. Heliyon 7, e07890. doi: 10.1016/j.heliyon.2021.e07890 34522801 PMC8427248

[B59] Padulosi S. Ng N. Q. (1997). “Origin, taxonomy, and morphology of Vigna unguiculata (L.) Walp,” in advances in cowpea research. Eds. Singh B. B. Mohan Raji D. R. Dashiel K. E. (IITA, Ibadan, Nigeria), 1–12.

[B60] Pasquet R. S. (1998). Morphological study of cultivated cowpea Vigna unguiculata (L.) Walp. Importance of ovule number and definition of Cv Gr Melanophthalmus. Agronomy 18, 61–70. doi: 10.1051/agro:19980104

[B61] Pasquet R. S. (2000). Allozyme diversity of cultivated cowpea Vigna unguiculata (L.)Walp. Theor. Appl. Genet. 101, 211–219. doi: 10.1007/s001220051471

[B62] Paudel D. Dareus R. Rosenwald J. Muñoz-Amatriaín M. Rios E. F. (2021). Genome-wide association study reveals candidate genes for flowering time in cowpea (Vigna unguiculata [L.] Walp.). Front. Genet. 12, 667038. doi: 10.3389/fgene.2021.667038 34220944 PMC8242349

[B63] Prasanthi L. Geetha B. Jyothi B. N. R. Reddy K. R.. (Eds.). (2012). Evaluation of genetic diversity in cowpea, Vigna unguiculata (L.) Walp gentotypes using Random Amplified Polymorphic DNA (RAPD). CURRENT BIOTICA 6, 22–31.

[B64] R Core Team (2021). R: A Language and Environment for Statistical Computing (Vienna, Austria: R Foundation for Statistical Computing). Available at: https://www.R-project.org/ (Accessed February 20, 2024).

[B65] R Core Team (2023). R: A Language and Environment for Statistical Computing (Vienna, Austria: R Foundation for Statistical Computing). Available at: https://www.R-project.org/ (Accessed November 5, 2024).

[B66] Santos S. P. D. Araújo M. D. S. Aragão W. F. L. D. Damasceno-Silva K. J. Rocha M. D. M. (2024). Genetic analysis of yield component traits in cowpea [Vigna unguiculata (L.) Walp. Crop Breed. Appl. Biotechnol. 24, e46432413. doi: 10.1590/1984-70332024v24n1a03

[B67] Sarr A. Bodian A. Gbedevi K. M. Ndir K. N. Ajewole O. O. Gueye B.. (2021). Genetic diversity and population structure analyses of wild relatives and cultivated cowpea (Vigna unguiculata (L.) walp.) from Senegal using simple sequence repeat markers. Plant Mol. Biol. Rep. 39, 112–124. doi: 10.1007/s11105-020-01232-z

[B68] Seo E. Kim K. Jun T.-H. Choi J. Kim S.-H. Muñoz-Amatriaín M.. (2020). Population structure and genetic diversity in korean cowpea germplasm based on SNP markers. Plants (Basel Switzerland) 9, 1190. doi: 10.3390/plants9091190 32932572 PMC7569878

[B69] Stoilova T. Pereira G. (2013). Assessment of the genetic diversity in a germplasm collection of cowpea (Vigna unguiculata (L.) Walp.) using morphological traits. Afr. J. Agric. Res. 8, 208–215. doi: 10.5897/AJAR12.1633

[B70] Timko M. P. Rushton P. J. Laudeman T. W. Bokowiec M. T. Chipumuro E. Cheung F.. (2008). Sequencing and analysis of the gene-rich space of cowpea. BMC Genomics 9, 103. doi: 10.1186/1471-2164-9-103 18304330 PMC2279124

[B71] Uffelmann E. Huang Q. Q. Munung N. S. De Vries J. Okada Y. Martin A. R.. (2021). Genome-wide association studies. Nat. Rev. Methods Primers 1, 59. doi: 10.1038/s43586-021-00056-9

[B72] Walle T. Mekbib F. Amsalu B. Gedil M. (2018). Correlation and path coefficient analyses of cowpea (Vigna unguiculata L.) landraces in Ethiopia. Am. J. Plant Sci. 9, 13. doi: 10.4236/ajps.2018.913202

[B73] Walle T. Mekbib F. Amsalu B. Gedil M. (2019). Genetic diversity of Ethiopian cowpea [Vigna unguiculata (L) Walp] genotypes using multivariate analyses. Ethiopian J. Agric. Sci. 29, 89–104.

[B74] Wu X. Michael V. N. López-Hernández F. Cortés A. J. Morris J. B. Wang M.. (2024). Genetic diversity and genome-wide association in cowpeas (Vigna unguiculata L. Walp). Agronomy 14, 961. doi: 10.3390/agronomy14050961

[B75] Xavier A. Xu S. Muir W. M. Rainey K. M. (2015). NAM: association studies in multiple populations. Bioinformatics 31, 3862–3864. doi: 10.1093/bioinformatics/btv448 26243017

[B76] Xiong H. Shi A. Mou B. Qin J. Motes D. Lu W.. (2016). Genetic diversity and population structure of cowpea (Vigna unguiculata L. Walp). PloS One 11, e0160941. doi: 10.1371/journal.pone.0160941 27509049 PMC4980000

[B77] Yin L. Zhang H. Tang Z. Xu J. Yin D. Zhang Z.. (2021). rMVP: a memory-efficient, visualization-enhanced, and parallel-accelerated tool for genome-wide association study. Genomics Proteomics Bioinf. 19, 619–628. doi: 10.1016/j.gpb.2020.10.007 PMC9040015 33662620

