# Supplementary figures and images for: Cowpea genetic diversity, population structure and genome-wide association studies in Malawi: insights for breeding programs
Source: Front Plant Sci. 2025 Jan 20;15:1461631. doi: 10.3389/fpls.2024.1461631 (PMC11788394; doi:10.3389/fpls.2024.1461631)

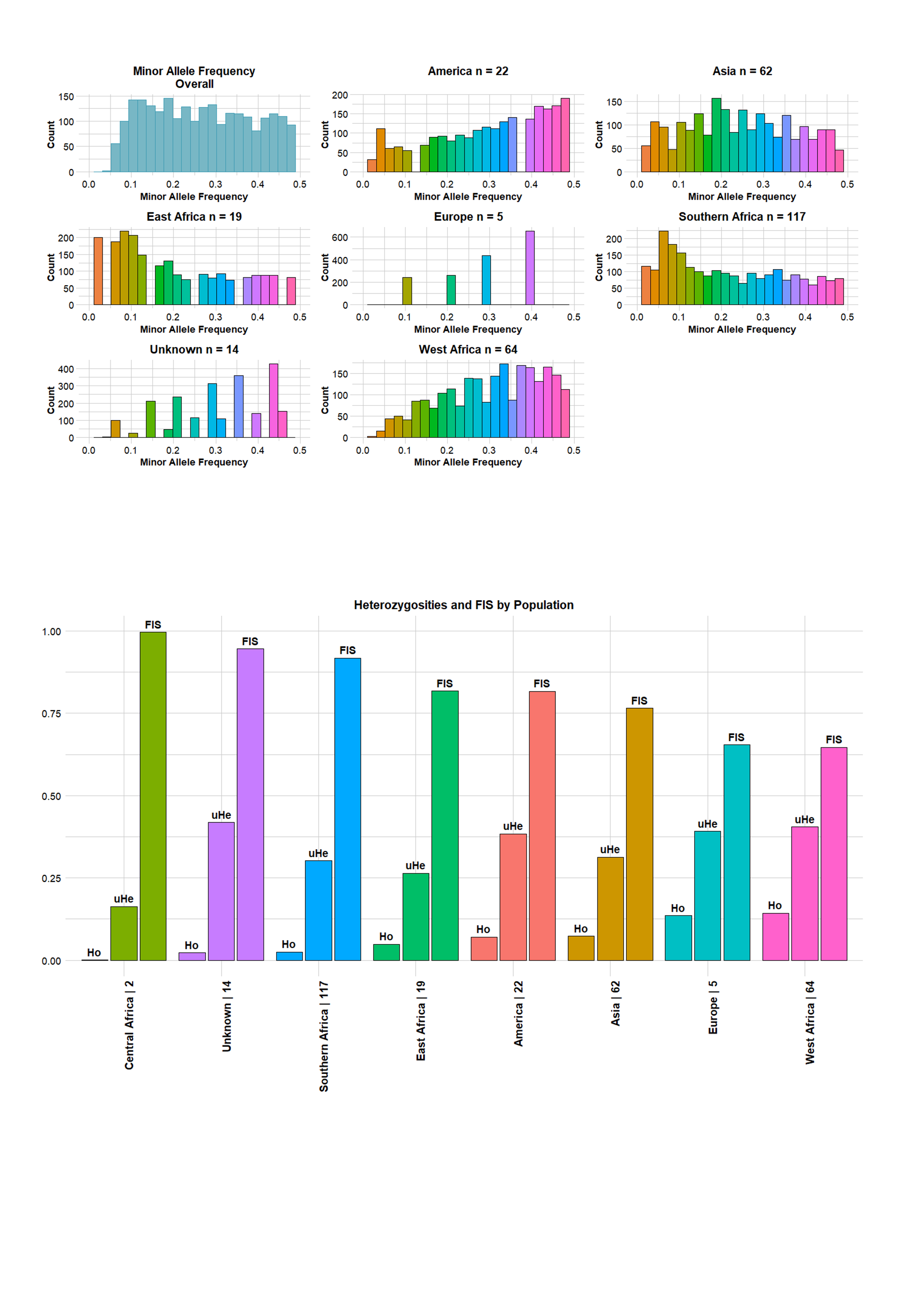

Supplement: Supplementary file 1 [file Image1.png]

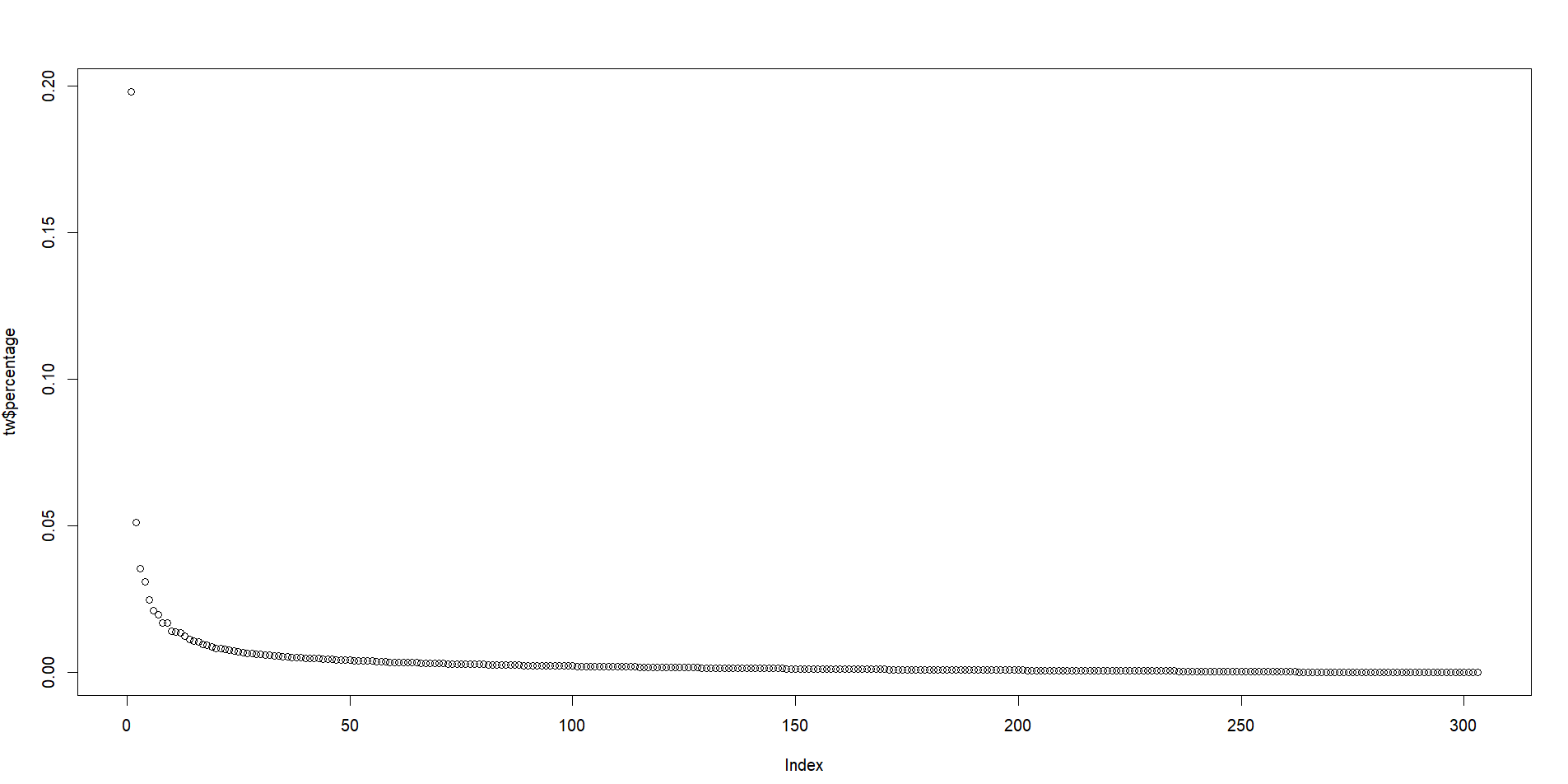

Supplement: Supplementary file 2 [file Image2.png]

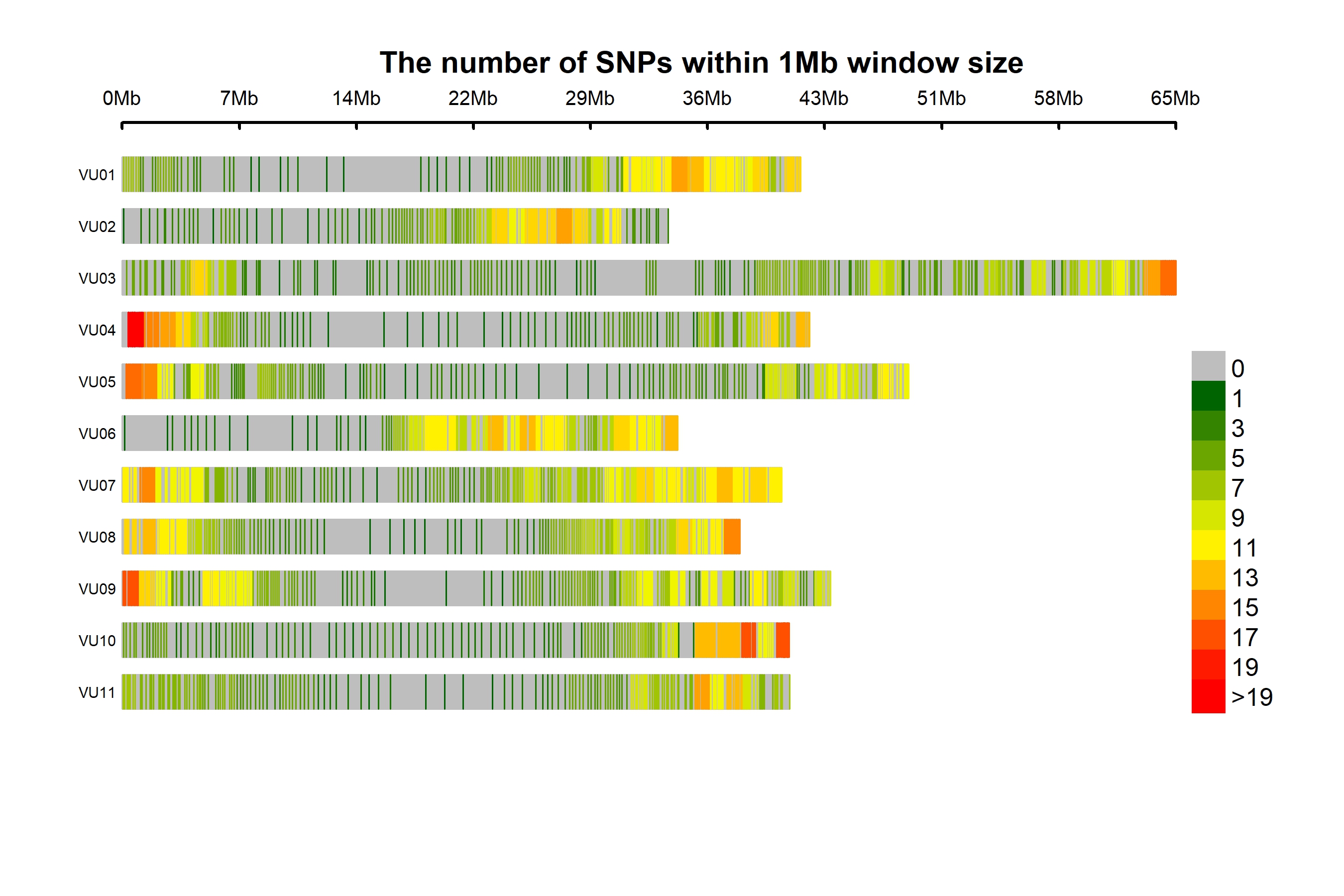

Supplement: Supplementary file 3 [file Image3.jpeg]
